# Mechanically Adaptative and Environmentally Stable Ionogels for Energy Harvest

**DOI:** 10.1002/advs.202300253

**Published:** 2023-04-21

**Authors:** Wei Zhao, Zhouyue Lei, Peiyi Wu

**Affiliations:** ^1^ State Key Laboratory for Modification of Chemical Fibers and Polymer Materials College of Chemistry and Chemical Engineering Center for Advanced Low‐Dimension Materials Donghua University Shanghai 201620 China; ^2^ John A. Paulson School of Engineering and Applied Sciences Harvard University Cambridge MA 02138 USA

**Keywords:** energy harvesting, environmental tolerance, ionogels, mechanical adaptability, synergistic ionic associations

## Abstract

Converting building and environment heat into electricity is a promising strategy for energy harvest to tackle global energy and environmental problems. The processing challenges, mechanical brittleness, and low environmental tolerance of typical thermoelectric materials, however, prevent them from realizing their full potential when employed in outdoor building systems. Herein, a general concept based on synergistic ionic associations to significantly improve the mechanical properties and harsh environment stability for high‐performance ionic‐type thermoelectric (i‐TE) gels is explored. They demonstrate extraordinarily high stretchability (1300–2100%), fast self‐healing (120 s), temperature insensitivity, and great water‐proof performance, and could be painted on a variety of surfaces. The n‐type ionic Seebeck coefficient is up to −8.8 mV K^−1^ and the ionic conductivity is more than 0.14 mS cm^−1^. Both exhibit remarkable thermal and humidity stability (293–333 K, 20–100 RH%), which are rarely achieved in previous studies. Even on a cloudy day, the open‐circuit thermovoltage for a painted i‐TE array with an area of about 8.5 × 10^−3^ m^2^ is above 2 V. This research offers a promising approach for gathering significant waste heat and even solar energy on outside building surfaces in an effective and sustainable manner.

## Graphical abstract

Ionogels designed by synergistic ionic associations enable to harvest hybrid low‐grade thermal and solar energy for energy‐saving buildings. They exhibit superior mechanical adaptability and environmental stability (293–333 K, 20–100 RH%) with a boosted thermovoltage. The open‐circuit voltage for a painted array with an area of less than 0.01 m^2^ is higher than 2 V even on an overcast day.

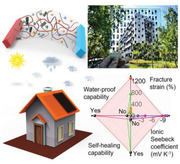

## 1 Introduction

The need for high‐performance and reasonably priced materials for energy conversion is growing due to concerns about global warming and energy consumption.^[^ ^1^, ^2^, ^3^ ^]^ A significant portion of the world's energy is used by buildings, and significant waste heat losses exacerbate the global warming challenge.^[^ ^4^ ^]^ One of the most promising building components for energy collection and conservation is the roofing system. On the one hand, passive radiative coatings are created for daytime cooling in order to lessen the net heating effect.^[^ ^5^, ^6^, ^7^ ^]^ On the other hand, materials that can directly convert heat into electricity are receiving a lot of attention as a promising strategy to utilizing waste heat and addressing the world's energy concerns.^[^ ^6^, ^8^, ^9^, ^10^ ^]^ If ordinary building coatings are inexpensively changed to thermoelectric coatings, they could significantly increase the utilization of building waste heat and even solar energy, while alleviating the energy and environmental crisis.

Traditional thermoelectric materials, however, couldn't be used as coatings for energy‐efficient buildings. Due to their intrinsic fragility, inorganic thermoelectric materials made of semiconductive components are challenging to prepare for coatings across a large surface area.^[^ ^11^ ^]^ Mechanical flexibility is a benefit of organic conductive polymers, however they typically have a low Seebeck coefficient (S_e_ < 1 mV K^‐1^).^[^ ^12^, ^13^ ^]^ Recently, some ionotronic materials have emerged as promising candidates due to the thermodiffusion of ions (Soret effect) in a temperature field. They are known as ionic‐type thermoelectric (i‐TE) materials and often exhibit a high ionic Seebeck coefficient, particularly a high p‐type Seebeck coefficient (*S_i_* > 1 mV K^−1^, the subscript *i* indicates the ionic type instead of the electric type).^[^ ^14^, ^15^, ^16^, ^17^, ^18^, ^19^, ^20^, ^21^ ^]^ Unfortunately, two grand challenges severely limit the applications of i‐TE materials. 1) Ionotronic materials with high ionic Seebeck coefficients exhibit poor mechanical properties, in contrast to ordinary ionic conductors.^[^ ^18^, ^22^, ^23^, ^24^, ^25^ ^]^ Young's modulus and stretchability are typically below 0.1 MPa and 1000%, respectively.^[^ ^18^, ^24^ ^]^ 2) The majority of the current i‐TE materials are hydrogels or ionogels with significant hygroscopicity, which causes them to expand in rain and dehydrate in sunlight.^[^ ^16^, ^18^, ^21^, ^22^, ^24^, ^26^ ^]^ As a result, their shapes and thermoelectric performance are extremely unstable in outdoor environments. In particular, rare reports demonstrate a high n‐type ionic Seebeck coefficient at low humidity below 40%.^[^ ^18^, ^27^, ^28^, ^29^ ^]^

In this work, the above challenges are tackled by a general concept of synergistic ionic associations in i‐TE materials. Commercial polyacrylate coatings are upgrade by introducing hydrophobic ionic liquids and 2D nanosheets (MXene) to develop i‐TE coatings. There are synergistic ionic associations among the polymer, cations of ionic liquids (ILs), and electronegative nanosheets. Even at a very high temperature (333 K) and low humidity of 20%, the cations are selectively confined in physically crosslinked networks, resulting in amplified n‐type Soret effect (>−8 mV K^−1^). Besides, the ionogel shows tunable viscoelasticity for processing, mechanical adaptability at irregular interfaces, and outstanding stability under sun and rain washing. It could be painted on buildings to supply electricity by harvesting solar and waste heat energy.

## 2 Results and Discussions

### 2.1 Synergistic Ionic Association Design for the Ionogels

**Figure 1:**
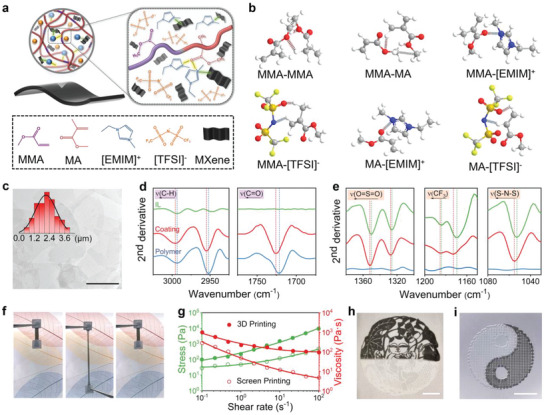
Synergistic ionic association design for the ionogels. a) Schematic illustration of the synergistic ionic associations among the polymer, cations of ILs, and MXene nanosheets. The dashed lines on the yellow and green background are ion‐dipole and electrostatic interactions, respectively. b) DFT calculations of molecule structures. c) Transmission electron microscopy (TEM) image of the exfoliated MXene sheets. The inset photograph is the probability distribution curves of their lateral sizes. Scale bar: 2 µm. Second derivative curves of d) the polymer and e) [TFSI]^−^ anions from the ionogel. f) Photographs of tensile resilience of the ionogel during a manual stretch‐release process. g) Shear‐thinning behavior of the ionogel precursor inks for screen printing and 3D printing by the rheology test. Photographs of customizable h) screen‐print pattern on the fabrics and i) 3D‐printing pattern on polypropylene membranes from the ionogel inks with and without MXene. Scale bar: 1 cm.

The schematic illustration of the design of the ionogel is shown in **Figure** **1**a. We use two common monomers from commercial polyacrylate coatings, that is, methyl methacrylate (MMA) and methyl acrylate (MA), to fabricate an entangled polymer network P(MMA‐*co*‐MA).^[^ ^30^, ^31^ ^]^ MMA and MA as the “hard monomer” and “soft monomer” contribute to solid‐like elasticity and liquid‐like viscosity, respectively.^[^ ^32^ ^]^ When the mass ratio of MMA and MA monomer is varied from 55:0, 53:2, 50:5 to 47:8, the modulus decreases accompanied by the increase of the stretchability (Figure S1, Supporting Information). A hydrophobic IL (1‐ethyl‐3‐methylimidazoliumbis (trifluoromethylsulfonyl) imide, [EMIM][TFSI]) is selected as the ionic charge carrier for the upgraded coating. Compared with previously reported hydrophilic ILs, it not only provides better environmental tolerance but also has strong ion‐dipole interactions with the polymer P(MMA‐*co*‐MA) to construct physically‐crosslinked networks.^[^ ^33^, ^34^ ^]^ The interaction forces between the IL and polymer chains are studied by density functional theory (DFT) calculations at the *ω*B97X‐D level using the 6–31 g(d) basis set (Figure 1b, Figure S2, and Table S1, Supporting Information).^[^ ^35^, ^36^ ^]^ The attractive binding energy between the PMMA segment and [EMIM]^+^ cation is calculated to be 475.56 kcal mol^−1^, which is much higher than that between the PMMA segment and [TFSI]^−^ anion (112.07 kcal mol^−1^). While the PMA segment is thermodynamically favorable to bind the [TFSI]^−^ anion (300.45 kcal mol^−1^) over the [EMIM]^+^ cation (54.76 kcal mol^−1^). To boost the negative Seebeck coefficient for the ionogel, we need to enlarge the diffusion difference between the cation and the anion by selectively trapping the cation in the dipole‐ion interacted networks according to the Soret effect. Thus, we choose a relatively high mass ratio (50:5) between the MMA and MA units. The ionic association between the polymer P(MMA‐*co*‐MA) and IL is dominated by cation‐dipole interactions. When increasing the amount of the IL, the ionogel's modulus decreases and the elongation at break increases (Figure S3, Supporting Information). But excessive IL leads to poor mechanical properties and even potential leakage of the ionic electrolyte. Here we choose an optimal polymer: IL mass ratio of 4.0:6.0. The ionogel shows a high transmittance of about 94% at the visible range, confirming the good miscibility of IL and the polymer via a strong ion‐dipole interaction (Figure S4, Supporting Information).^[^ ^34^ ^]^

To further enlarge the diffusion difference between the cation and the anion, electronegative and photothermally‐responsive MXene nanosheets are introduced in the ionogel. The MXene nanosheets are designed with a relatively large lateral size of several micrometers (Figure 1c and Figure S5, Supporting Information). The high surface area provides abundant electronegative terminal groups (such as —F, —OH, —O, and —Cl).^[^ ^37^, ^38^ ^]^ They tend to bind the [EMIM]^+^ cation and thus synergistically boost the negative ionic Seebeck coefficient. Upon the application of a temperature or photothermal difference, the synergistic ionic associations among the polymer, IL and MXene promote thermodiffusion of [TFSI]^−^ anions over [EMIM]^+^ cations, thus conferring a boosted n‐type Soret effect on the ionogel.^[^ ^19^, ^39^ ^]^

The synergistic ionic associations among the polymer, IL and MXene are further evidenced by Fourier Transform infrared (FTIR) spectra (Figure 1d,e, Figure S6, Note S1, and Table S2, Supporting Information). With the addition of IL, the aliphatic C—H stretching bands (2993 and 2952 cm^−1^) and C = O stretching band (1722 cm^−1^) of P (MMA‐*co*‐MA) shift to higher wavenumbers (Figure 1d). This indicates that IL destroys strong dipole‐dipole interactions in the polymer but enhances the ion‐dipole interactions between the polymer and IL. Meanwhile, the —O = S = O— antisymmetric bending, —CF_3_ bending, and —SNS— antisymmetric bending bands of the [TFSI]^−^ anion shift to higher wavenumbers (Figure 1e). They suggest the interactions between [TFSI]^−^ anion and [EMIM]^+^ cation is weakened due to the thermodynamically favorable cation‐dipole interaction of [EMIM]^+^‐polymer.^[^ ^40^ ^]^ With the introduction of electronegative MXene nanosheets, the C—H stretching band (3123 and 3098 cm^−1^) of the imidazole ring of the [EMIM]^+^ cation shifts to higher wavenumbers, indicating the electrostatic interaction of [EMIM]^+^‐MXene (Figure S6c, Supporting Information). Therefore, the synergistic ionic associations among the polymer, IL and MXene enlarge the diffusion difference between the cation and the anion, which could boost the n‐type Soret effect. Furthermore, the synergistic ionic associations construct solid‐like elastic networks for ionogels. The ionogel shows fast recoverability and negligible residual strain upon continuous loading‐unloading cycles (Figure 1f, Figure S7, and Video S1, Supporting Information).

When a small amount of organic solvent, for example, acetone, is added, the ionogel shows shear‐thinning behavior and tunable viscosity (Figure 1g and Figure S8, Supporting Information). It is liquid‐like under a high shear force and could retain its shape after the removal of the force. It can be facilely processed and directly used to paint various substrates with customized patterns. In particular, the solutions are suitable for screen‐printing and 3D printing with micrometer resolution. A variety of customized patterns (e.g., new year painting doll and Tai ji shapes) composed of different inks can be screen‐printed and 3D printed on different substrates like fabrics or polypropylene films (Figure 1h,i and Figures S9 and S10, Supporting Information). They demonstrate the great potential for large‐scale production and processing of the ionogel.

### 2.2 Mechanical Adaptability and Remarkable Stability of the Ionogels

**Figure 2:**
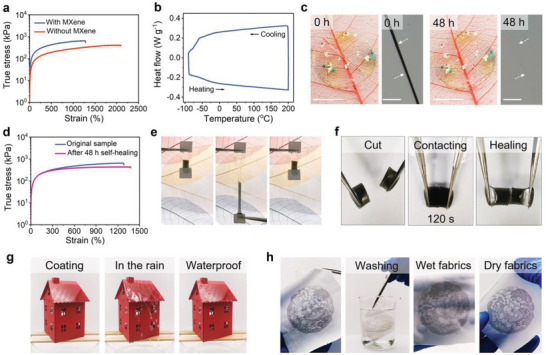
Mechanical adaptability and remarkable stability of the ionogels. a) True tensile stress–strain curves of the ionogels with and without MXene. b) Differential scanning calorimetry (DSC) curve of the ionogel in a temperature range of −90–200 °C. c) Photographs and optical micrographs of the autonomously self‐healing process of the MXene‐free ionogel. Scale bar: 1 cm and 500 µm, respectively. d) True tensile stress–strain curves of the original and healed ionogel with MXene. e) Photographs of tensile resilience of the assembled ionogel during a manual stretch‐release process. f) Photographs of the underwater self‐healing process of the ionogel. g) Hydrophobicity photographs of the coating on a model roof. h) Underwater stability photographs of screen‐print “new year painting doll” pattern on the fabrics.

As a result of the rational design of synergistic ionic associations, the ionogels show excellent mechanical properties. Without MXene, the ionogel shows an elongation at break of 2100% and Young's modulus of 0.178 MPa. With the addition of MXene, the elongation at break slightly decreases to 1300% while Young's modulus increases to 0.436 MPa (**Figure** **2**a). Young's modulus and stretchability are much higher than those of current thermoelectric materials.^[^ ^16^, ^18^, ^20^, ^24^, ^26^ ^]^ During compression tests, when the compressive strain increases to about 90%, the stress of the ionogels with MXene and without MXene is 4.57 and 3.72 MPa, respectively. (Figure S11, Supporting Information). Both ionogels keep the physical integration even at such high strain. Moreover, the ionogels are highly stable in extremely cold and hot environments. With or without the addition of MXene, there is no melting point and a glass transition of about −10 °C observed in the ionogels (Figure 2b and Figure S12, Supporting Information). The polymer networks will neither be completely frozen at 0 °C nor be destroyed at 100 °C, because they maintain the solid‐like elasticity with a higher storage modulus (*G*’) than loss modulus (*G*’’), as further confirmed by dynamic thermomechanical analysis (DMA) and rheological measurements (Note S2, Figures S13 and S14, Supporting Information). It is ascribed to remarkable thermal stability and negligible vapor pressure of the hydrophobic IL. Compared to aqueous i‐TE materials, our ionogels demonstrate their potential to be used in a harsh environment.

Besides, the combination of the lower glass transition temperature and highly reversible ionic associations endows the ionogels with the capability to self‐heal autonomously at room temperature.^[^ ^35^ ^]^ The self‐healing process of the MXene‐free ionogel in the air is recorded by time‐dependent photographs and optical micrographs (Figure 2c and Figure S15, Supporting Information). Within 48 h, a crack completely disappears. The self‐healing ionogels fully restore mechanical properties including the high Young's modulus (0.186–0.433 MPa) and ultra‐high stretchability (1380–2200%) (Figure 2d and Figure S16, Supporting Information). Moreover, two different pieces of the ionogels could be seamlessly assembled due to the excellent self‐healing capacity. The assembled sample is elastic and stretchable (Figure 2e and Video S1, Supporting Information). Even underwater, the cracked sample with or without MXene could also quickly heal the fractured interface in only 120 s (Figure 2f, Figure S17, and Video S2, Supporting Information). The self‐healing samples remain shape stability and mechanical stretchability. The underwater self‐healing capacity is because of the hydrophobicity of the polyacrylates and fluorine‐rich ILs. Water molecules minimally perturb the ion‐dipole and electrostatic interactions in the bulk and fractured interface of the sample.^[^ ^34^, ^41^ ^]^ The ionogel is thus water‐proof and highly stable underwater. When the ionogel is painted on a model roof surface, it also shows excellent water‐proof capability. The continuous artificial rainfall does not wash away the coating (Figure 2g and Video S3, Supporting Information). When the ionogel is screen‐printed on the fabric, the patterns neither dissolve nor swell under continuous soaking and washing even 7 days (Figure 2h, Figures S18 and S19, and Video S3, Supporting Information). When the ionogel is coated on A4 paper, the covered region remains undamaged and unpolluted with the dyed water even after 48 h underwater (Figures S20 and S21 and Video S3, Supporting Information). Furthermore, the ionogel is also highly stable even upon exposure to sunlight for 120 min. In a comparison, a i‐TE hydrogel shrinks in only 10 min due to the water evaporation (Figure S22, Supporting Information). Overall, the ionogel is not only mechanically adaptative in a wide temperature range, but also perfectly stable under rain and sunlight. These excellent properties render it promising for high‐efficient building energy conversion. Notably, we choose the 0.04 wt% of MXene because excessive MXene decreases the mechanical stretchability and self‐healing capacity of the ionogel.

### 2.3 Thermoelectric Properties and Robustness of the Ionogels

**Figure 3:**
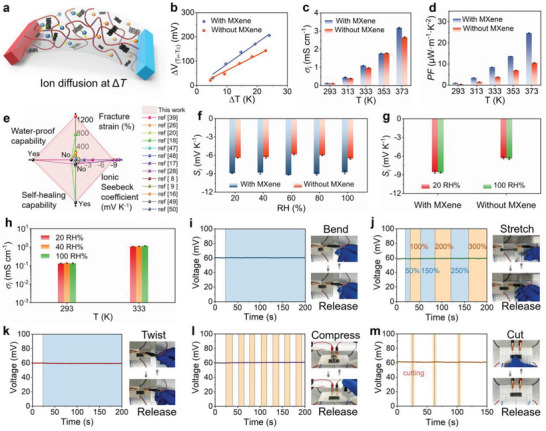
Thermoelectric properties and robustness of the ionogels. a) Schematic illustration of the ion thermodiffusion in the ionogel under a temperature difference. b) Potential difference at hot and cold terminals (Δ*V*_(TH‐TC)_) of the ionogel with (blue spots) or without MXene (orange spots) under different temperature gradients (Δ*T*). The slope of the Δ*V*_(TH‐TC)_‐Δ*T* curve is the absolute value of the ionic Seebeck coefficient (*S_i_*). c) Ionic conductivity (*σ_i_*) and d) power factor (*PF*) of the ionogel with (blue bars) or without MXene (orange bars) at different temperatures. The *PF* is calculated based on *S_i_* and *σ_i_* values obtained above. Data are presented as mean ± SD, *n* = 3. e) A comparison between this work and previously‐reported i‐TE materials in terms of stretchability, ionic Seebeck coefficient, self‐healing, and water‐proof capability. f) *S_i_* of the ionogel with (blue bars) or without MXene (orange bars) at different relative humidities. Data are presented as mean ± SD, *n* = 3. g) *S_i_* of the ionogel at high temperatures (333 K) and relative humidities (20–100 RH%). Data are presented as mean ± SD, *n* = 3. h) *σ_i_* of the ionogel in wide temperature and humidity ranges (293–333 K, 20–100 RH%). Data are presented as mean ± SD, *n* = 3. i) Voltage–time (blue) curve of the ionogel being repeatedly step‐bent and a manual bend‐release photograph. The blank area of the graph is in standby state. j) Voltage–time (green) curve of the ionogel being manually stretched from 50% to 300% strain and a manual stretch‐release photograph. k) Voltage–time (red) curve of the ionogel being repeatedly twisted and a manual twist‐release photograph. l) Voltage–time (purple) curve of the ionogel being repeatedly compressed‐released by a 20 g load, which is recorded by a photograph. m) Voltage–time (orange) curve of the ionogel being repeatedly cut by a sharp knife and a manual cut‐release photograph.

When the ionogel is painted on a building and located in a temperature gradient, ionic charge carriers in the ionogel diffuse according to the Soret effect and attract complementary electrons at the electrode interface. The Seebeck coefficient (*S_i_*) of ions, similar to the thermoelectrics, can be defined as the ratio between the electric field −*dV*/*dx* and the temperature gradient *dT*/*dx*, and can be further written as:

(1) $$S_{i}=\;-\;\frac{dV/dx}{\;dT/dx}=\;-\frac{V\left(T_{H}\right)-V\left(T_{C}\right)}{T_{H}-T_{C}}$$

where *V* is the voltage, *T*_H_ and *T*_C_ are the temperatures at the hot electrode and the cold electrode, respectively.^[^ ^16^ ^]^ The anions easily diffuse from the hot side to the cold side over the cations, when a temperature difference occurs in the ionogel (**Figure** **3**a). As previously demonstrated by DFT and FTIR spectra, the polymer and MXene have a higher binding capability with cations over anions due to the synergistic ionic associations, hindering the movement of the cations.^[^ ^19^, ^39^ ^]^ The *S_i_* is measured on a self‐made temperature gradient platform (Figure S23 and Note S3, Supporting Information). The ionogel exhibits a larger *S_i_* of −8.8 mV K^−1^ compared with the MXene‐free ionogel of −6.3 mV K^−1^ (Figure 3b). The ionic conductivity (*σ_i_*) of the ionogels is obtained by the electrochemical impedance spectroscopy (Note S4, Supporting Information).^[^ ^22^ ^]^ When the temperature is raised from 293 to 373 K, *σ_i_* of the ionogel significantly increases from 0.14 to 3.18 mS cm^−1^, which is higher than the MXene‐free ionogel from 0.12 to 2.66 mS cm^−1^ (Figure 3c). These phenomena suggest that MXene enlarges the diffusion difference between cations and anions, and also increases the concentration of ion carriers. The increase of ion concentration is attributed to the dissociation of associated ion species in [EMIM][TFSI], due to the electrostatic interaction between MXene and [EMIM]^+^ cations. Moreover, the electronic conductivity (*σ_e_*) of the ionogels with and without MXene is measured by a polarization current‐time test at room temperature (293 K) (Note S4, Supporting Information).^[^ ^42^, ^43^ ^]^ As shown in Figure S24, Supporting Information, the *σ_e_* value of the ionogels with MXene is calculated to be 3.43 × 10^−6^ mS cm^−1^, which is comparable to that of the MXene‐free ionogels (1.72 × 10^−6^ mS cm^−1^). The *σ_i_* value of the MXene‐added ionogels (0.14 mS cm^−1^) is about five orders of magnitude higher than the *σ_e_* value. Therefore, the ionogels with MXene is an ionic conductor. The power factor (*PF* *= S* ^2^ *σ*) is generally used to evaluate the thermoelectric properties of the polymer materials, and it increases with rising temperature due to an enhanced *σ_i_* (Figure 3d).^[^ ^18^, ^24^, ^44^, ^45^ ^]^ With the increased *σ_i_* and *S_i_*, the *PF* of the MXene‐added ionogel is significantly higher than that of the MXene‐free ionogel (25.4 vs 10.6 µW m^−1^ K^−2^ at 373 K). The *PF* value is comparable to that of conventional i‐TE materials (Table S3, Supporting Information). With the increase of IL content, the *S_i_* and *σ_i_* can be further improved to enhance the *PF* value (Figures S25 and S26, Supporting Information). But as mentioned above, excessive IL leads to poor mechanical properties and environmental stability, and even potential leakage of the ionic electrolyte. The thermal conductivity (*λ*) of the ionogel is measured based on a modified transient line source method (Note S5 and Figure S27, Supporting Information). At room temperature, the *λ* values of the ionogels with and without MXene are 0.1930 and 0.1949 W m^−1^ K^−1^, respectively. They are quite low and comparable to the thermal conductivity of the pure polymer (0.091 W m^−1^ K^−1^) and IL (0.141 W m^−1^ K^−1^).^[^ ^22^ ^]^ Thermal conduction is related to the lattice vibration, and the discontinuation between two phases can lower the thermal conductivity.^[^ ^46^ ^]^ The ionic figure of merit (*ZT = S* ^2^ *σT/λ*) of the MXene‐added ionogel is thus estimated to be 0.05.

Compared with existing i‐TE materials (including ionogels,^[^ ^18^, ^20^, ^26^, ^39^ ^]^ ionic conducting polymers,^[^ ^17^, ^28^, ^47^, ^48^ ^]^ ionic quasi‐solid and liquid thermoselectrochemical cells,^[^ ^8^, ^9^, ^16^, ^49^, ^50^ ^]^ etc.), our ionogel shows distinct advantages in terms of excellent water‐proof capability, autonomous self‐healing, extremely high stretchability, and large negative Seebeck coefficient (Figure 3e and Table S3, Supporting Information). Existing ionic materials are usually p‐type, hygroscopic, limited by small elongation at break, and lack of self‐healing ability in extreme conditions.^[^ ^16^, ^18^, ^20^, ^26^ ^]^ Rare materials are reported for such a large n‐type ionic Seebeck coefficient and combine the advantages of mechanical adaptability and remarkable stability.^[^ ^17^, ^28^, ^39^, ^51^, ^52^ ^]^ Specially, in contrast to previous thermoelectric ionogels/hydrogels, our ionogels show stable *S_i_* and *σ_i_* in a wide range of humidity (20–100 RH%), as shown in Figure S28 and Table S4, Supporting Information.^[^ ^18^, ^20^, ^26^, ^28^, ^47^, ^53^ ^]^ The change of *S_i_* is less than 7% when the humidity decreases from 100 to 20 RH% (Figure 3f and Figures S28–S30, Supporting Information). The ionogel with the addition of MXene remains to be −8.8 mV K^−1^ at the low humidity of 20%. In contrast, the *S_i_* of a hydrophilic i‐TE material decreases from 8.1 to 1.3 mV K^−1^ when the humidity decreases from 90 to 50 RH% due to inadequate dynamic interaction.^[^ ^18^ ^]^ Meanwhile, the ionic conductivity of the ionogel is also highly stable. There is only a 10% change in the *σ_i_* when the humidity decreases from 100 to 20 RH% (Figures S28 and S31, Supporting Information). While the drop is up to 99% for the conventional hydrophilic materials.^[^ ^18^ ^]^ In addition to the stable performance at room temperature, even at a high temperature of 333 K, the *S_i_* and *σ_i_* of our ionogels remain highly stable. The change of *S_i_* is within 3% when the humidity decreases from 100 to 20 RH% (Figure 3g and Figures S32 and S33, Supporting Information). The ionogel without MXene has only 1.5% change in the *S_i_*. Meanwhile, the *σ_i_* of the ionogel is also highly stable, only with a change below 7% (Figure 3h and Figure S34, Supporting Information). This is benefited from the remarkable thermal stability and hydrophobicity of the ionogels.

We further demonstrate the robustness of the ionogels during dynamic deformation (Figure 3i–m, Note S6, and Video S4, Supporting Information). Under a temperature gradient of 7 K, the open‐circuit thermovoltages of about 61.4 and 44.5 mV are generated for the ionogels with and without MXene, respectively (Figure S36, Supporting Information). When the ionogel is stepped bent (with a bending angle of 90°) repeatedly, it can maintain stable thermovoltages (Figure 3i and Figure S37, Supporting Information). When the ionogel is stretched from 50 to 300% strain, the open‐circuit thermovoltages remain stable during each manual stretch‐release process (Figure 3j and Figure S38, Supporting Information). When the ionogel is twisted repeatedly, the thermovoltages are well‐preserved (Figure 3k and Figure S39, Supporting Information). Then, when the ionogel is compressed‐released (residence time of about 10 s for each operation) repeatedly by a 20 g load, the open‐circuit thermovoltages remain stable (Figure 3l and Figure S40, Supporting Information). Moreover, when the ionogel is repeatedly cut by a sharp knife, the thermovoltages fluctuate slightly but immediately return to the initial value due to its excellent self‐healing ability (Figure 3m and Figure S41, Supporting Information).

### 2.4 The Applications of Ionic‐Type Thermoelectric Capacitor

**Figure 4:**
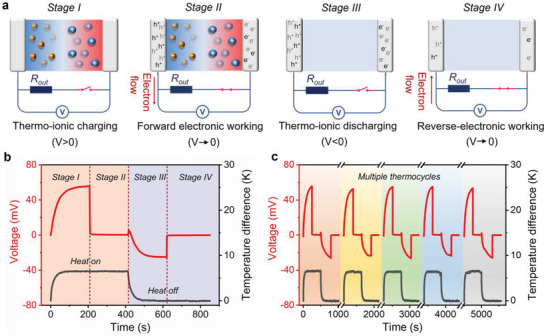
The application of the i‐TE capacitor. a) Schematic illustration of the working principle of the i‐TE capacitor. b) Voltage (red line) and temperature (grey line) curves of the i‐TE capacitor with an external load of 20 kΩ under a cycle. c) Voltage (red line) and temperature (grey line) curves of the i‐TE capacitor under multiple continuous cycles.

An i‐TE capacitor is then assembled with the same configuration as the ionic Seebeck coefficient measurement device but connected to the external circuit with a load (Note S7, Supporting Information). Two symmetric platinum wires are used as electrodes. The i‐TE capacitor works in a quasi‐continuous mode (**Figure** **4**a,b). Throughout stage I, when a temperature difference of about 6.4 K establishes across the i‐TE capacitor, [EMIM]^+^ cations and [TFSI]^−^ anions thermodiffuse and accumulate at electrodes to generate thermovoltage. As expected, the thermovoltage increases and reaches a stable value of about 55.6 mV after 207 s. Throughout stage II, an external load with a resistance of 20 kΩ is connected to the i‐TE capacitor. The electrical current can spontaneously flow through the external load to compensate for the charge imbalance between two electrodes. The thermovoltage decreases rapidly and finally reaches a stable value of about 0.18 mV after 208 s. This stage is referred to as the charging process of the i‐TE capacitor. Throughout stage III, the external load is disconnected, and the heat source is switched off. The disappearance of the temperature gradient will drive the accumulated cations and anions at electrodes to diffuse back to the original state. This causes the thermovoltage to gradually decay to zero. However, electrons remain on the electrode and induce a voltage drop to govern the open circuit potential. The voltage drop increases during the stage and reaches a stable value of about −25.1 mV after 208 s. This charged i‐TE capacitor can be utilized as a power source. Throughout stage IV, when the external load of 20 kΩ is connected again, electrons flow away from the capacitor electrodes and pass through the external electrode to do work. The electrical current flow in stage IV is opposite to that in stage II. These four stages can realize the energy conversion from thermal heat to electricity in stage II and stage IV. The charging and discharging process is repeatable and stable in multiple cycles (Figure 4c). This demonstrates not only the stability but also the true potential of the i‐TE capacitor for harvesting electricity from intermittent heat sources.

Notably, the ionic thermovoltage decays rapidly and reaches a relatively low output voltage in stage II, which is a typical characteristic of the ionic conductor due to their intrinsic capacitive feature.^[^ ^18^, ^26^ ^]^ Fortunately, one of the methods to improve the output voltage has been discussed in previous studies, such as introducing electrochemical reaction at the electrode.^[^ ^54^, ^55^ ^]^ The electrochemical reaction at the electrode can allow ions to enter into and exit from the polymer film to increase the duration time of output voltage. Therefore, the combination of the electrochemical reaction and the Soret effect is beneficial to improve the total output voltage and increase the working time.

### 2.5 The Scalable Integration of the Ionogels

**Figure 5:**
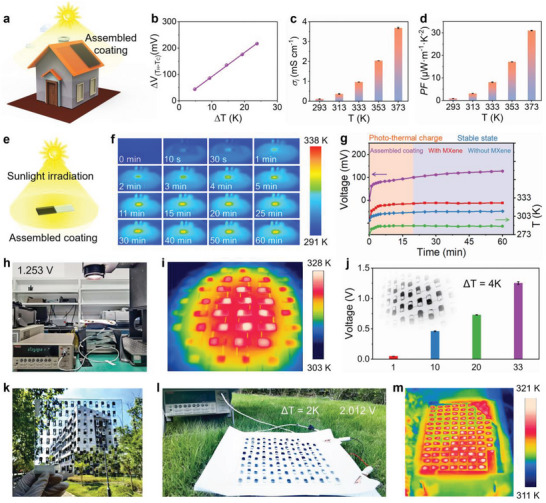
The self‐powered capacity and scalable integration of the ionogels. a) Schematic illustration of a self‐powered building coating via interfacial self‐healing. b) Potential difference at hot and cold terminals (Δ*V*_(TH‐TC)_) of the self‐powered coating under different temperature gradient Δ*T*. The slope of the Δ*V*_(TH‐TC)_‐Δ*T* curve is the absolute value of the ionic Seebeck coefficient (*S_i_*). c) Ionic conductivity (*σ_i_*) and d) power factor (*PF*) of the self‐powered coating at different temperatures. Data are presented as mean ± SD, *n* = 3. e) Schematic illustration of the thermophotovoltaic conversion of the self‐powered coating under one sun illumination (1 kW m^−2^). f) Infrared images of the self‐powered coating with different irradiation times. g) Changes of the surface temperatures of the MXene‐added side (hot end, red squares) and the MXene‐free side (cold end, blue diamonds), temperature difference (green spots), and generated thermovoltage (purple hexagons) under one sun irradiation. h) The voltage and i) infrared images of an integrated array containing 33 units under one sun illumination with moderate intensity about 0.3 kW m^−2^. j) The voltage of different coating arrays. Data are presented as mean ± SD, *n* = 3. The insert illustrates the connection of every assembled coating element. The size of every element is about 14.0 × 6.0 × 1.0 mm^3^. k) An integrated large array containing 101 units. l) The open‐circuit voltage and m) infrared images of the integrated large array in a cloudy environment.

To demonstrate the self‐powered capacity of the ionogel, two types of the ionogel is connected in series, in which one is transparent and the other is added with MXene. MXene is a perfect photothermal material with nearly 100% internal photothermal conversion efficiency. It could absorb solar energy at the wavelength of about 793 nm and then spontaneously establish a temperature gradient field in the ionogel (Figure S42, Supporting Information).^[^ ^38^, ^56^ ^]^ Therefore, it shows a self‐powdered capacity due to the photo‐thermal‐electricity conversion. As shown in **Figure** **5**a, when we assemble two pieces of the coating (with and without MXene) via interfacial self‐healing, it establishes a planar temperature gradient on the building roof upon exposure to sunlight. A large *S_i_* of about −9.2 mV K^−1^ is obtained from the assembled coating, which is even slightly higher than that of the pure ionogel with/without MXene (Figure 5b). This is because the heterogeneity of the composite coating further enlarges the difference of the ion diffusion, leading to an enhanced Soret effect. The *σ_i_* gradually increases to 3.69 mS cm^−1^ when the average temperature rises to 373 K (Figure 5c). The increased *S_i_* and *σ_i_* lead to an enhanced *PF* value of 31.2 µW m^−1^ K^−2^ (Figure 5d). We further investigate the thermoelectric voltage under artificial sun illumination (1 kW m^−2^) (Figure 5e). The surface temperature of the MXene‐added side increases from 296.3 to 320.4 K upon exposure to solar irradiation in about 5 min and reaches an equilibrium temperature of 324.9 K after 60 min. In contrast, the MXene‐free side rapidly increases from 296.3 to 306.7 K and then the equilibrium of 311.3 K (Figure 5f). During the sun illumination process, a stable thermovoltage of about 127.6 mV is gradually generated following a final temperature gradient Δ*T* of about 13.6 K between two terminals. Detailed changes of both terminal surface temperature and generated thermovoltage are shown in Figure 5g.

The scalable integration of electric generators is critical for realizing highly efficient power output in the ambient environment. The output voltage could be enhanced by proper connection. The assembled coatings are connected in series by silver electrodes and integrated into different arrays without extra encapsulation. The integrated modules could be directly applied to roofing systems and are highly stable in wide ranges of humidity and temperature (Figure 5h). Under a solar simulator with moderate intensity of 0.3 kW m^−2^ to mimic a cloudy environment, a spontaneous temperature difference of about 4 K is established (Figure 5i and Figure S43, Supporting Information). The thermovoltage increases from about 0.049 to 1.253 V when the thermoelectric coating assembles an array of about 33 elements (Figure 5j). When the coating is painted on a glass substrate, it tightly adheres to the substrate. Benefiting from the convenience of processing, it is easy to assemble a large array with 101 units (about 8.5  ×  10^−3^ m^2^, Figure 5k). In a cloudy outdoor environment, this array generates a stable open‐circuit voltage of up to 2.012 V at a temperature difference of only 2 K (Figure 5l,m). Natural solar energy and waste heat are ubiquitous, providing opportunities for the actual performance of power‐supplying systems in real conditions. The coating can produce considerable thermovoltage by spontaneously absorbing solar energy and harvesting low‐grade thermal sources over a wide range of temperatures (293–333 K) and humidity (20–100 RH%), adapting to most of the environmental scenarios on outdoor building surfaces. Notably, the photothermal property of the ionogel provides their surface with a faster self‐healing capability driven by illumination. This further updates the capability of the coating to deal with the physical damage and even chemical degradation (i.e., oxidation of MXene nanosheets), maintaining maximum thermoelectric performance.^[^ ^56^ ^]^

## 3 Conclusion

In summary, we demonstrate a general concept based on the synergistic ionic associations to construct high‐performance i‐TE gels. The mechanically adaptative, environmentally stable, and water‐proof i‐TE gel is facilely prepared from non‐solvent polymerization, revealing that the synergistic ionic associations can simultaneously fine modulate the processing, mechanical and electrical properties of i‐TE materials. It shows tunable viscoelasticity for processing, ultrahigh stretchability (1300–2100%), remarkable Young's modulus (0.178–0.436 MPa), and excellent mechanical adaptability to irregular surfaces, fast self‐healing capability (120 s), impressive stability under the rain, sunlight, and extremely high/cold temperatures. More importantly, the synergistic ionic associations effectively enlarge the diffusion difference between the anions and the cation in a temperature gradient. It leads to a boosted n‐type ionic Seebeck coefficient of −8.8 mV K^−1^ and ionic conductivity of 0.14 mS cm^−1^ at extremely low humidity, which has been rarely demonstrated in previous studies. The *ZT* of the ionogel is estimated to be 0.05. Furthermore, the i‐TE gels could spontaneously create a planar temperature gradient field after being painted on a building's roof. The electricity could be generated not only from waste heat dissipated by the building but also from solar energy. A stable open‐circuit voltage of more than 2 V is reached from an integrated large array containing 101 units on a cloudy day. This work presents a general strategy for the design of i‐TE gels, paving the way for the rational design of intrinsically stable and high‐performance i‐TE materials for building energy saving.

## 4 Experimental Section

A detailed Experimental Section can be found in Supporting Information.

## Conflict of Interest

The authors declare no conflict of interest.

## Supporting information

Supporting Information

Supplemental Video 1

Supplemental Video 2

Supplemental Video 3

Supplemental Video 4

## Data Availability

The data that support the findings of this study are available from the corresponding author upon reasonable request.
