# Supplementary material for: Mechanically Adaptative and Environmentally Stable Ionogels for Energy Harvest
Source: Adv Sci (Weinh). 2023 Apr 21;10(18):2300253. doi: 10.1002/advs.202300253 (PMC10288276; doi:10.1002/advs.202300253)
Supplement: Supplementary file 1 — Supporting Information [file ADVS-10-2300253-s003.pdf]

ADVANCED SCIENCE
Open Access

Supporting Information

for *Adv. Sci.*, DOI 10.1002/advs.202300253

Mechanically Adaptative and Environmentally Stable Ionogels for Energy Harvest

Wei Zhao, Zhouyue Lei and Peiyi Wu**

Supporting Information

Mechanically Adaptative and Environmentally Stable Ionogels for Energy Harvest

Wei Zhao, Zhouyue Lei, and Peiyi Wu**

W. Zhao, Z. Lei, P. Wu
State Key Laboratory for Modification of Chemical Fibers and Polymer Materials, College of Chemistry and Chemical Engineering, Center for Advanced Low-Dimension Materials, Donghua University, Shanghai, China, 201620.
E-mail: leizhouyue@dhu.edu.cn; wupeiyi@dhu.edu.cn.

Z. Lei
John A. Paulson School of Engineering and Applied Sciences, Harvard University, Cambridge, MA, 02138, USA.

Experimental Section

Materials: Methyl methacrylate (MMA) and Methyl acrylate (MA) monomers were purchased from TCI co (Shanghai). 1-ethyl-3-methylimidazoliumbis (trifluoromethylsulfonyl) imide ([EMIM][TFSI]) was bought from ALADIN co (Shanghai). Lithium fluoride (LiF) and 2-hydroxy-2-methylpropiophenone (HMPP) were obtained from Sigma-Aldrich co. Ti_3AlC_2 was purchased from 11 technology Co. Hydrochloric acid (HCl) and acetone were bought from Sinopharm Chemical Reagent Co. Platinum wires were used as electrodes. The high-adhesion dielectric tape VHB (4905) was obtained from Minnesota Mining and Manufacturing Co. Deionized water with a resistivity of 18.2 $M\Omega \cdot cm^{-1}$ was obtained from Millipore reverse osmosis water purification system.

Exfoliation of MXene: The MXene nanosheets were obtained by utilizing LiF/HCl solution to etch the aluminum layers in Ti_3AlC_2. Typically, 2.0 g LiF was added into 40 mL HCl aqueous solution (the volume ratio of HCl to deionized water of 3:1) under vigorous stirring to obtain the etching solution. Then, Ti_3AlC_2 powder (2.0 g) was added within 5 min and stirred magnetically in an ice bath. After that, the mixture solution was stirred at 35 ºC for 24 h. The etched multilayered MXene was collected via centrifugation at 3500 rpm for 5 min, and then washed with water until the pH of the supernatant was close to 7. Subsequently, the washed residue was dispersed in deionized water (200 mL) and ultrasonication for 1 h, and then centrifuged at 3500 rpm for 1 h. Finally, delaminated MXene nanosheets were obtained after the supernatant was freeze-dried.

Preparation of the ionogels: A typical procedure for preparing the ionogel without MXene was as follows: MMA and MA monomers were mixed with IL and then initiated by HMPP (0.1 mol% of monomers). First, the mass ratio of MMA and MA was fixed at 50:5, while the weight ratio of total monomer to IL varies, including 5.0:5.0, 4.5:5.5, 4.0:6.0 and 3.5:6.5. Then, the weight ratio of monomers to IL was fixed at 4.0:6.0, while the ratio of MMA to MA monomer was tuned by 55:0, 53:2, 50:5, and 47:8, respectively. After centrifugation for 0.5 h, the precursor solution was poured in a Teflon mold with a glass plate for sealing. The polymerization was initiated by the ultraviolet light and left to proceed for 3 h. The thickness of the MXene-free ionogel was about 1mm.

Preparing the MXene-added ionogel was as follows: 4.0 g of the MXene-free ionogel and 1.6 mg of MXene powders were dissolved in 4.0 mL acetone to form a black and homogeneous

solution. Then the solution was poured into a 7×3×4 cm^3 Teflon sink and placed at room temperature for 48 h to volatilize acetone completely. The thickness of the ionogel was about 1mm.

Preparation of the ionogel inks with and without MXene: Typically, both inks were obtained by dissolving the MXene-free ionogel into acetone with or without MXene nanosheets. A series of inks with different viscosity could be obtained by adjusting the ratio of the ionogel and acetone, and adapted to various processing methods.

Screen printing of the ionogel inks with and without MXene on the fabrics: A screen mesh was fixed onto the printed devices, then both inks were loaded onto the screen mesh and swiftly screen printed onto the substrate. The screen (100-300 mesh) was made of polyester fabrics and a coating layer of photosensitive adhesive (PLUS8000). The blade was made of polyurethane rubber with a shore hardness of 85. When the blade was applied to fabricate the printed pattern, a pressure of about 0.4 MPa was used. After drying only for 10 min at room conditions, the printed fabrics were obtained due to rapid evaporation of acetone solvent.

3D printing of the ionogel inks with and without MXene: A 3D printing system, a 3D Bio-Architect work station, Regenovo, was used to print both inks. The accompanying software was used to build the models for 3D printing. The tip needle with a diameter of 0.26 mm and the speed of 4 mm s^{-1} were chosen in the 3D printing process. The viscosity of the ink decreased due to the shear thinning effect during the extrusion process; when the shear force was removed and the solvent evaporated, the inks returned to elastic solid. The printing process was carried out at 25 °C.

Characterization: Differential scanning calorimetry (DSC) data were obtained using a TA Instrument (DSC 250) with a scan rate of 10 °C min^{-1} under a dry nitrogen environment. Dynamic thermomechanical analyzer (DMA) measurements were performed on a DMA Q800 by a stretching mode with a frequency of 1 Hz and a heating rate of 5 °C min^{-1}. A HAAKE MARS modular advanced rheometer with a 25 mm parallel plate was utilized to record Storage (G') and loss (G'') moduli at 50 and 100 °C with an oscillation mode at a fixed 0.5 % oscillatory strain. Viscosity was performed with the shear rates ranging from 0.1 to 100 s^{-1} at room temperature. Infrared spectra were collected using an attenuated total reflectance Fourier transform infrared spectroscopy (ATR-FTIR, Nicolet iS50 spectrometer) under atmospheric

conditions. Transmittance and absorbance of the ionogel were recorded on a SHIMADZU UV-Vis spectrophotometer (UV 2600) with scanning wavelengths from 300 to 900 nm. Morphology of the MXene nanosheets was characterized using a transmission electron microscope (TEM, JEM-2100 at 200 kV). Tensile curves were recorded on a universal mechanical test machine (UTM 2103) at ambient conditions. Here, the true stress instead of the nominal stress was used due to large deformation of the sample. The stress-strain curves were recorded at a strain speed of 50 mm min^{-1}. During compression tests, the compressive stress-strain curves were recorded at a strain speed of 50 mm min^{-1}. The thickness and the diameter of the sample were about 4 and 19 mm, respectively. An optical microscope (OLYMPUS SZX7) was used to record the self-healing process of the MXene-free ionogel in real-time. The self-powered coating was irradiated by a solar simulator (Newport 94043A, Class AAA) with an AM 1.5 light filter. The optical power density was measured and calibrated using a standard Si solar cell (Newport 91150). And the surface temperatures of the ionogels with and without MXene were detected by an infrared thermal camera (Testo 872 or FLIR ONE Pro) with an accuracy of ± 1 ^{o}C. The voltage and ionic conductivity were recorded on an Autolab potentiostat/galvanostat instrument (Metrohm, MAC 90510) at about 40% RH. The ionic Seebeck coefficient measurement was illustrated in Note S3 and Figure S23. The ionic conductivity (σ_i) was determined by the electrochemical impedance spectra (EIS), and the electronic conductivity (σ_e) was measured by a polarization current-time test, which were shown in Note S4. The thermovoltage of the integrated array was recorded by a nanovoltmeter (Keithley 2182A). The constant temperature and humidity chamber (BINDER) was used to maintain the humidity of the ionogels to obtain the conductivity and thermoelectric properties under different humidity conditions. The thermal conductivity was tested on a XIATECH Thermal Conductivity Analyzer (TC 3200) and shown in Note S5.

Note S1. FTIR analysis of the synergistic ionic associations among the polymer, ILs and MXene

The synergistic ionic associations among the polymer, IL and MXene are evidenced by FTIR spectra (Figure 1d-e and Figure S6). With the addition of IL, the aliphatic C-H stretching bands (2993 and 2952 cm^{-1}) and C=O stretching band (1722 cm^{-1}) of P (MMA-co-MA) shift to higher wavenumbers (Figure 1d). This indicates that IL destroys strong dipole-dipole interactions in the polymer but enhances the ion-dipole interactions between the polymer and IL. Meanwhile, the -O=S=O- antisymmetric bending, -CF_3 bending, and -SNS- antisymmetric bending bands of the $[TFSI]^-$ anion shift to higher wavenumbers (Figure 1e). They suggest the interactions between $[TFSI]^-$ anion and $[EMIM]^+$ cation is weakened due to the thermodynamically

favorable cation-dipole interaction of polymer-$[EMIM]^+$. With the introduction of electronegative MXene nanosheets, the C-H stretching band (3123 and 3098 cm^{-1}) of imidazole ring of the $[EMIM]^+$ cation shifts to higher wavenumbers, indicating the electrostatic interaction of MXene-$[EMIM]^+$ (Figure S6c). Therefore, the synergistic ionic associations among the polymer, IL and MXene enlarge diffusion difference between the cation and the anion, which could boost n-type Seebeck effect. Furthermore, the synergistic ionic associations construct solid-like elastic networks for ionogels. The detailed IR band migrations are presented in Table S2.

Note S2. DSC, DMA results and rheological behaviors of the ionogels

There is no melting point and glass transition of about -10 ºC observed in DSC curves of ionogels whether or not it contains MXene sheets. This indicates that there is no free water, and the IL in our material does not crystallize even at a temperature as low as -10 ºC. Thus, the IL is effectively bonded by polymer networks to provide good environmental stability. Interestingly, there is a glass transition observed in the DSC curves below -10 ºC, while a very broad glass transition region is shown in the DMA curves (Figure S13). It is worthwhile to note that, the glass transition temperature in DMA curves is determined by the shapes of the curves of the storage modulus and the loss modulus. There is no plateau region of the storage modulus curve and no peak of the loss modulus curve in our material. This suggests that the polymers' local segmental motion is not completely frozen even at a temperature as low as -10 ºC. In another word, there are always different modes of molecular motion that are active in such a broad region. Furthermore, as shown in the rheological results (Figure S14), the polymer networks maintain the solid-like elasticity at 50 and 100 ºC, since the storage modulus (G') is always higher than the loss modulus (G''). Therefore, the ionogel is anti-freezing and also stable at high temperatures, confirmed by the DSC, DMA, and rheology measurements.

Note S3. Ionic Seebeck coefficient measurement

The ionic Seebeck coefficient (S_i) of the ionogel is measured by a self-made temperature gradient platform (Figure S23). Two commercial Peltier elements are used as a thermal stage and a cold stage to generate the temperature difference. The VHB tapes (4905, 3M Company) are used for electrical insulation, and facilitate stability tests of thermoelectric behavior during multiple deform-release processes later. Platinum wires with a diameter of about 0.2 mm are used as electrodes. Two thermocouples are attached to the hot and cold terminals by the VHB tapes and kept in the same plane as platinum electrodes. The heat sink is used to maintain a

stable temperature in hot or cold side. The location of ionogel is symmetrical about the center of temperature field. The open-circuit voltage is recorded on an Autolab potentiostat/galvanostat (Metrohm, MAC90510).

Note S4. Electrochemical measurements

The ionic conductivity (σ_i) of the ionogels is determined by the electrochemical impedance spectra (EIS) on an Autolab potentiostat/galvanostat (Metrohm, MAC90510)[1]. The voltage amplitude is 5 mV, and the frequency is from 100 kHz down to 0.1 Hz. The ionogels are sandwiched between two stainless steel plates. The resistance (R) is obtained by extrapolating the curve with the abscissa. Then, the σ_i value is calculated from the equation,

$\sigma_i = L/(R\times S)$

where L is the film thickness (0.1 cm), and S is the film area (2 cm^2).

The electronic conductivity (σ_e) of ionogels is measured by the polarization current-time curves using symmetrical stainless steel cell.[2, 3] The σ_e value is calculated from the following Equation,

$\sigma_e = (I\times L)/(U\times S)$

where I is the steady-state current, L is the film thickness (0.1 cm), U is the polarization voltage (1 V), and S is the film area (2 cm^2).

Note S5. Thermal conductivity measurement

The thermal conductivity is tested on a XIATECH Thermal Conductivity Analyzer (TC 3200). It is based on a transient heating method, i.e., detecting the in-line thermal conductivity based on a linear transient heater of 2.5×1.0 cm^2. The linear transient heater is sandwiched tightly between two ionogels, and they are then squeezed in both quartz bricks for thermal conductivity tests. To improve the accuracy, each sample is measured at least three times. The rectangular sample (20×10×1 mm^3) is used for the measurement.

Note S6. Robustness measurement of thermoelectric performance

The effect of various deformations on the thermoelectric performance of the ionogels is evaluated by a self-made temperature gradient platform, which varies for different deformation operations. For bend, stretch, and twist deformations, the temperature gradient platform consists of the ambient environment (293 K) and a Peltier heating element (300 K) (Figure S35). The ionogels with and without MXene can continually generate a stable voltage of about 61.4 and 44.5 mV, respectively (Figure S36). For subsequent compress and cut operations, the temperature gradient platform is the ionic Seebeck coefficient measurement configuration. It is

composed of Peltier heating and cooling elements with a same temperature difference ΔT about 7 K. Specially, during the compression operation, the thin and soft plastic is used to isolate the ionogels from the metal load. The thickness of the ionogel is about 1 mm.

Note S7. Fabrication of i-TE capacitor

The i-TE capacitor is assembled with the same configuration as the ionic Seebeck coefficient measurement device but connected to the external circuit with a load. A large resistor (ZX21g) with a range of 1 to 10^8 Ω is connected to two platinum wire electrodes. The separation distance between two platinum electrodes is 1 cm. The thermovoltages are recorded in real-time with an Autolab potentiostat/galvanostat (Metrohm, MAC90510).

Videos

Video S1. Stretch-release process of the ionogels.
Video S2. Underwater self-healing capability of the ionogels.
Video S3. Hydrophobicity and underwater stability of the ionogels.
Video S4. Stability of the thermovoltage of the ionogels during deformation.

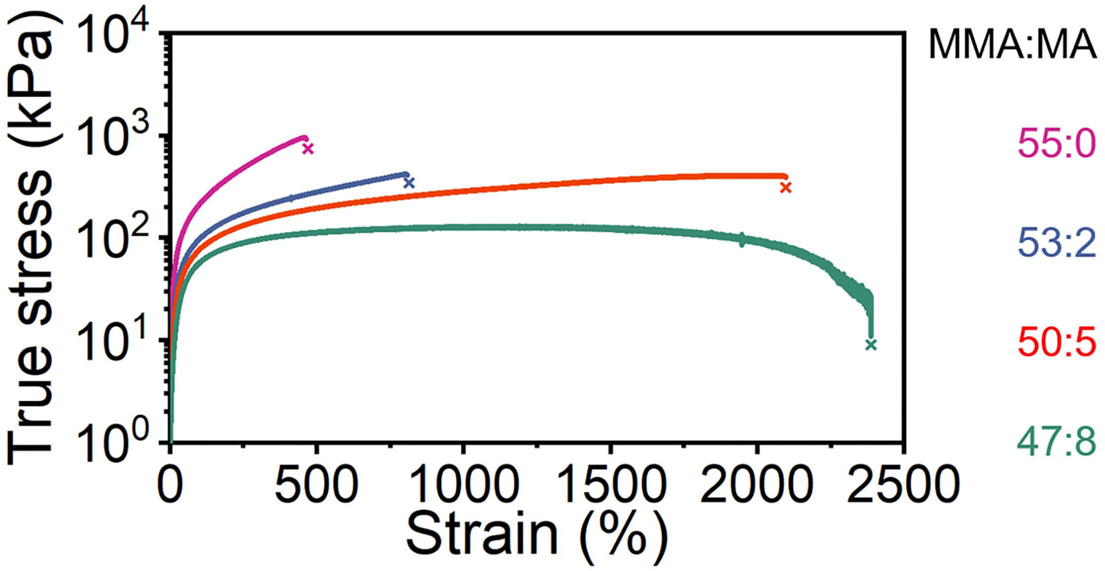

Figure S1. True tensile stress-strain curves of the MXene-free ionogel with different mass ratios of MMA to MA monomer.

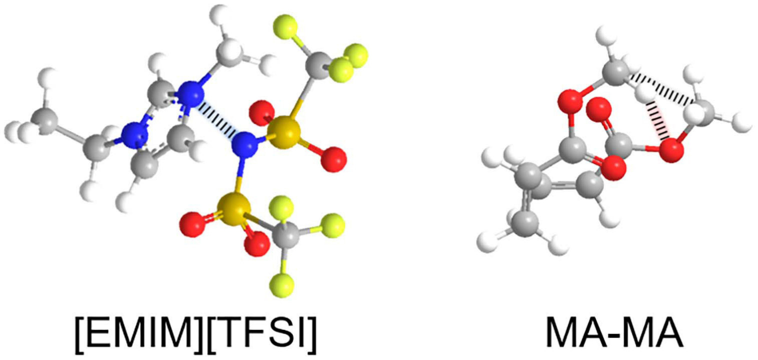

Figure S2. DFT calculations of molecule structures.

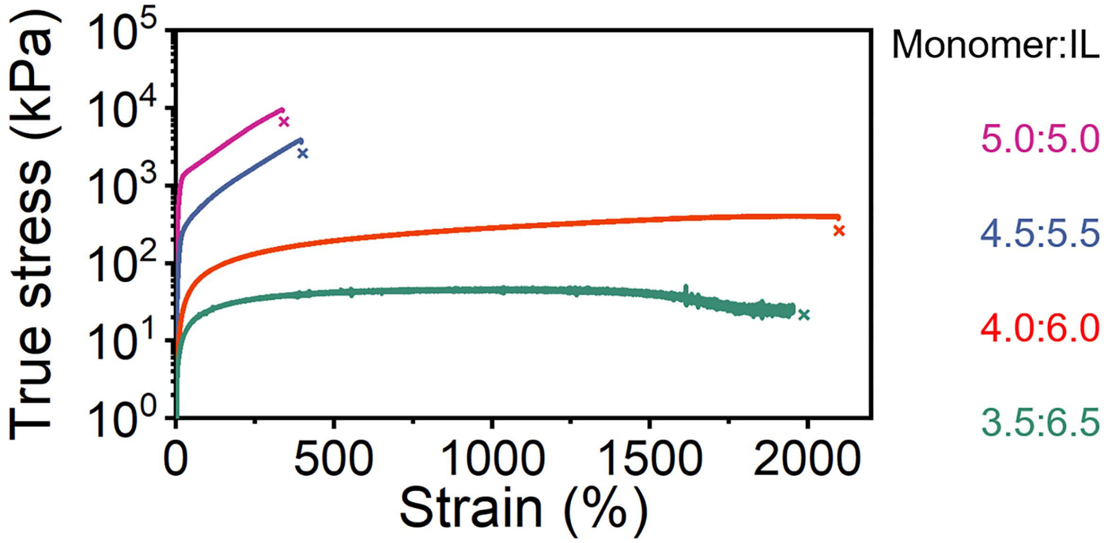

Figure S3. True tensile stress-strain curves of the MXene-free ionogel with different weight ratios of monomer to IL.

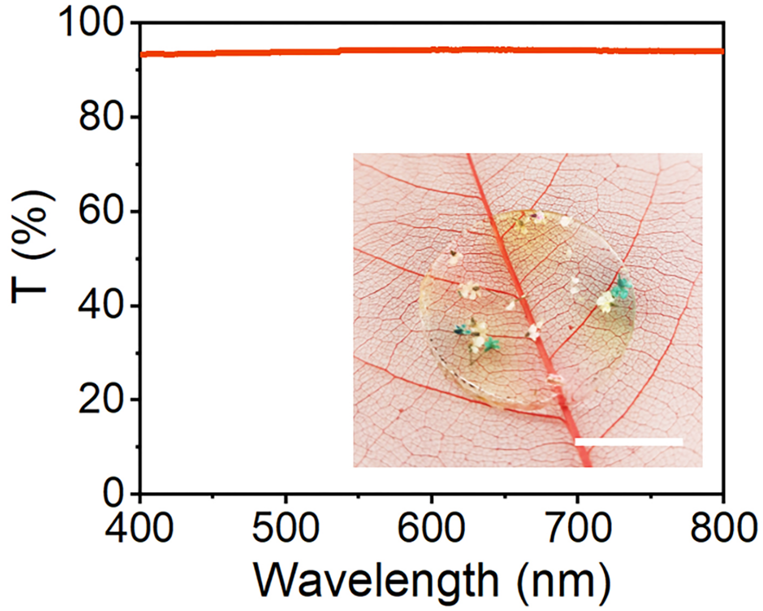

Figure S4. The transmittance of the MXene-free ionogel with a thickness of 1mm in the visible wavelength range of 400-800 nm. The inset is a photograph of a round sample on a piece of leaf. Scale bar: 1 cm.

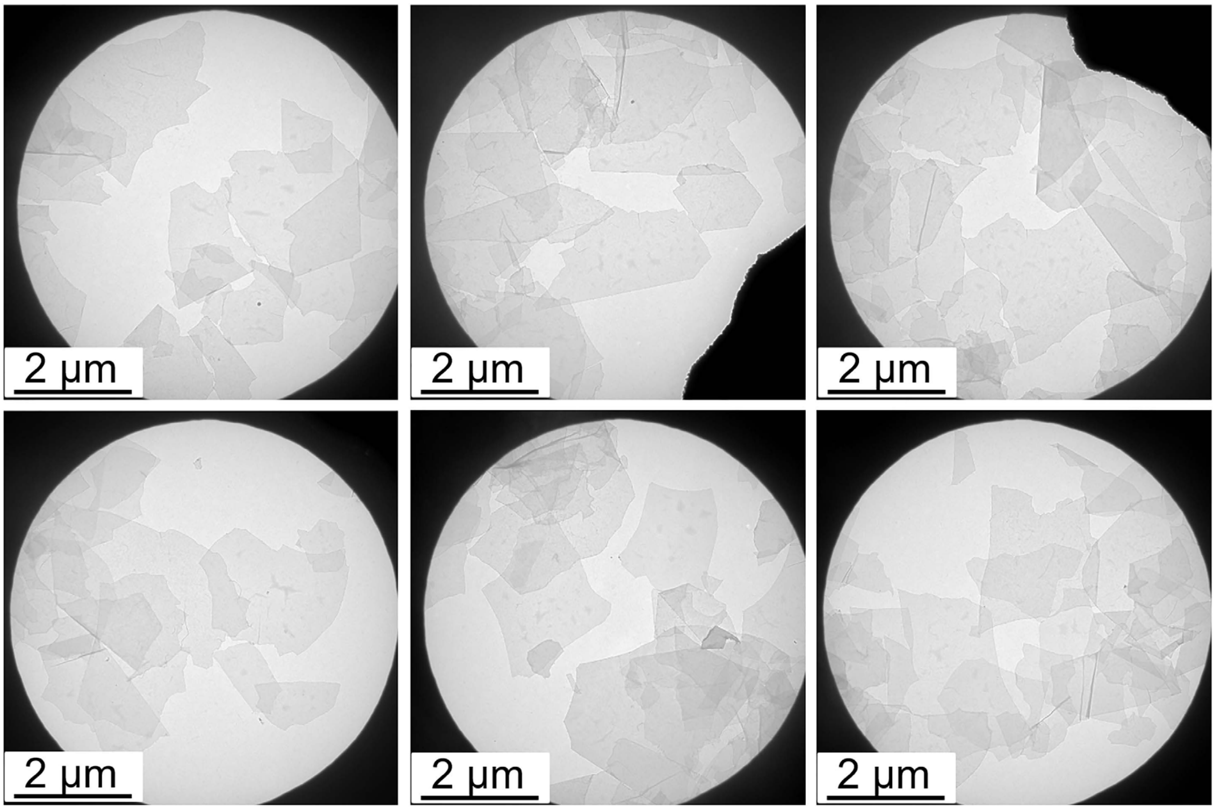

Figure S5. TEM image of the exfoliated MXene sheets.

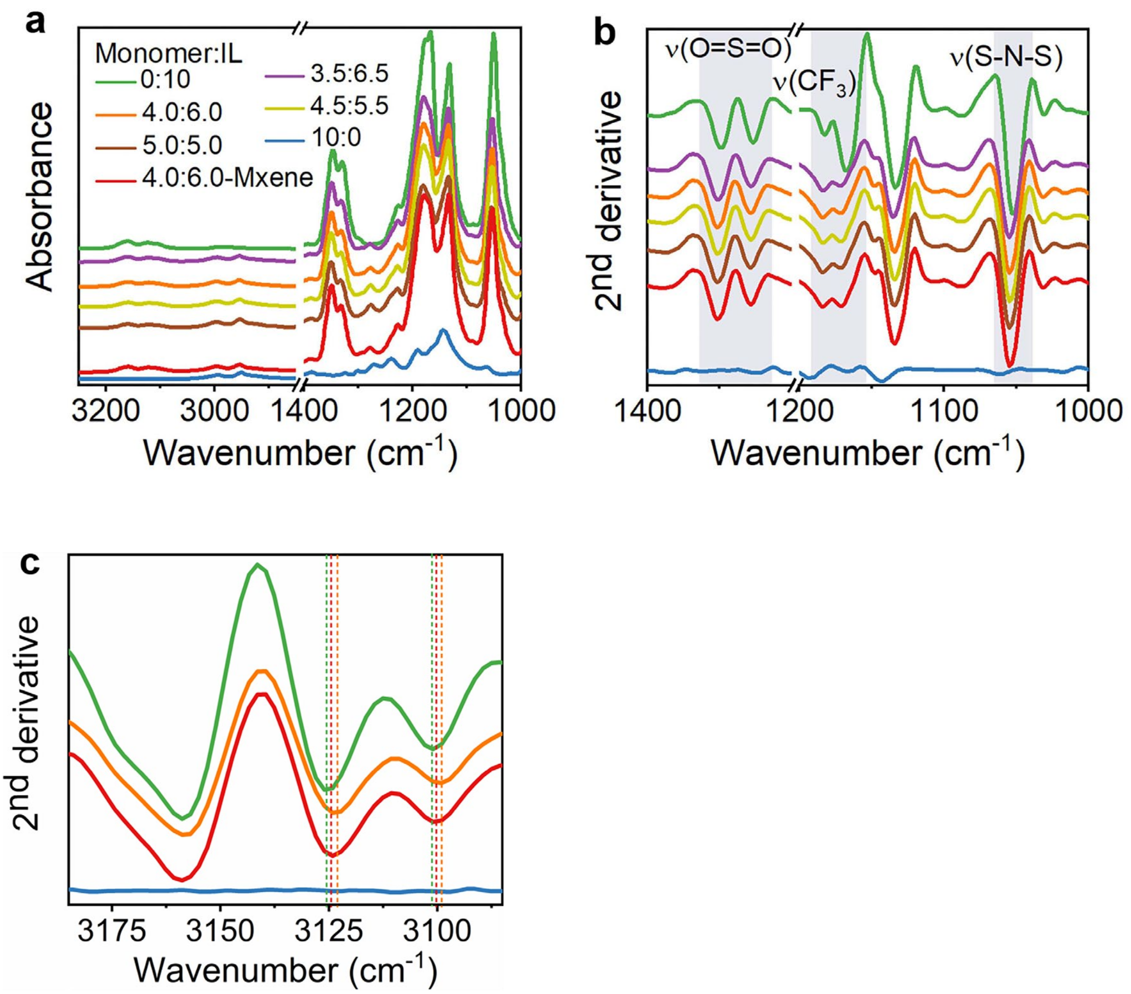

Figure S6. (a) FTIR spectra of the ionogels with different mass ratios of monomer to IL and (b) corresponding second derivative curves of the $[TFSI]^{-}$ anion in the region of 1400-1000 cm^{-1}. (c) Second derivative curves of the $[EMIM]^{+}$ cation in the region of 3185-3085 cm^{-1}.

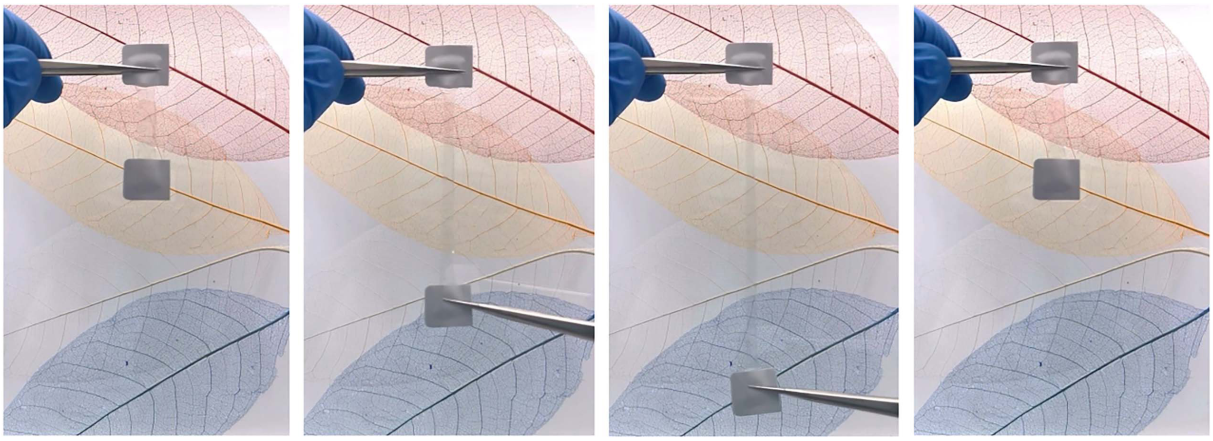

Figure S7. Photographs of tensile resilience of the MXene-free ionogel with a content of 60 wt% IL and an MMA:MA mass ratio of 50:5 during a manual stretch-release process. The recovery time of maximum elongation is about 90 s.

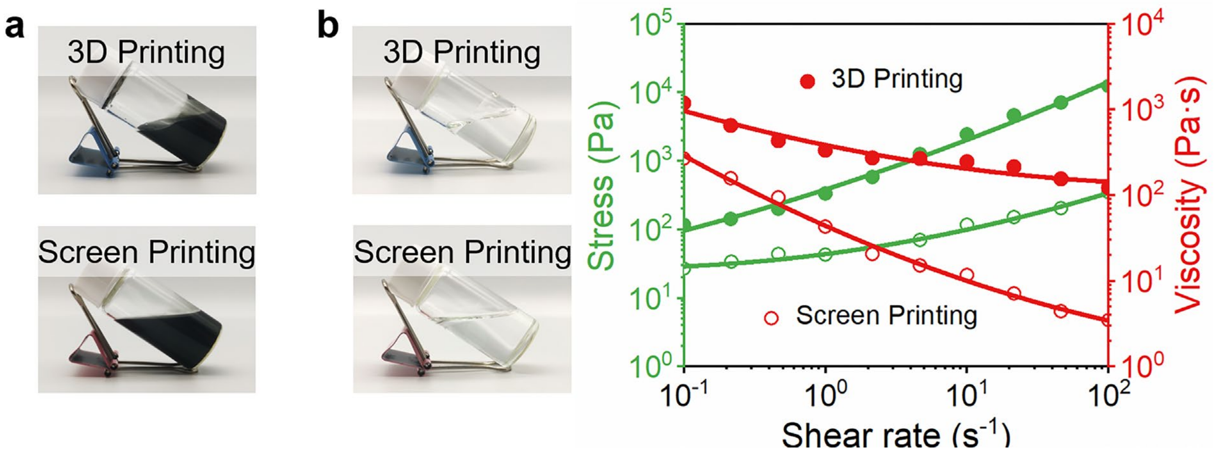

Figure S8. (a) Photographs of printing ink with MXene and (b) without MXene and its shear-thinning behaviors for screen printing and 3D printing by the rheology test.

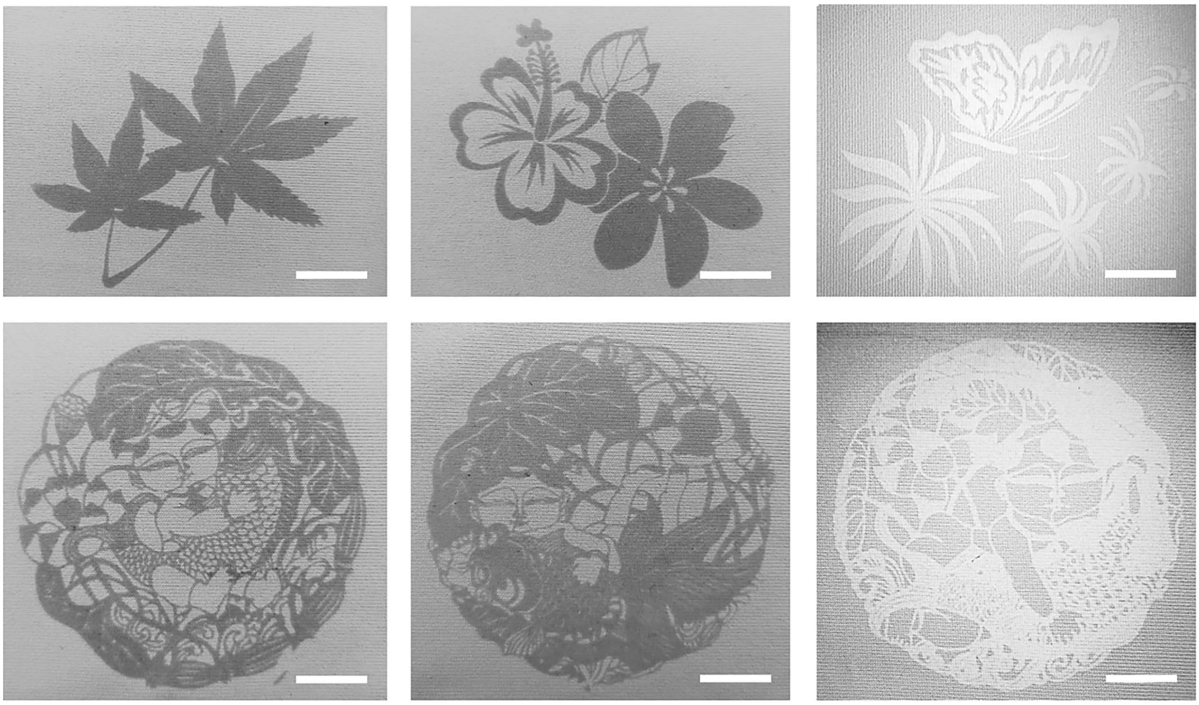

Figure S9. Photographs of customizable screen-print patterns from both ionogel inks on the fabrics. Scale bar: 1 cm.

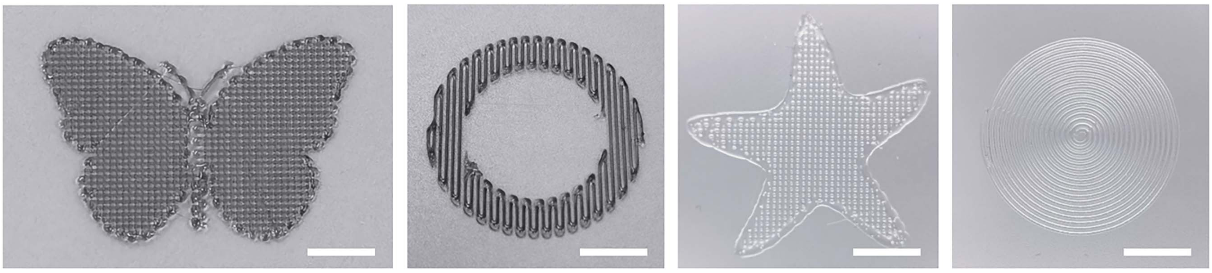

Figure S10. Photographs of customizable 3D-printing patterns from both ionogel inks on polypropylene membranes. Scale bar: 1 cm.

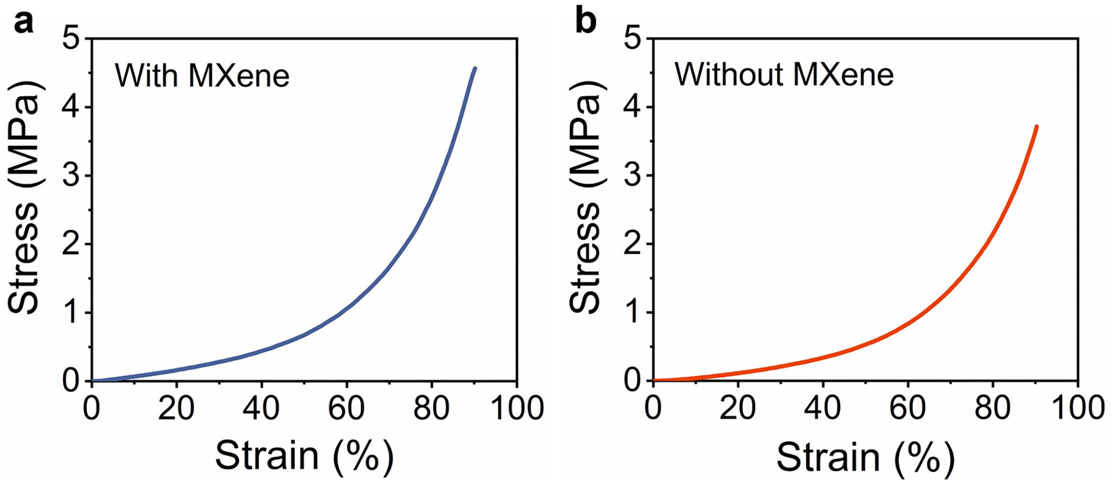

Figure S11. The compressive stress-strain curves of the ionogels with and without MXene.

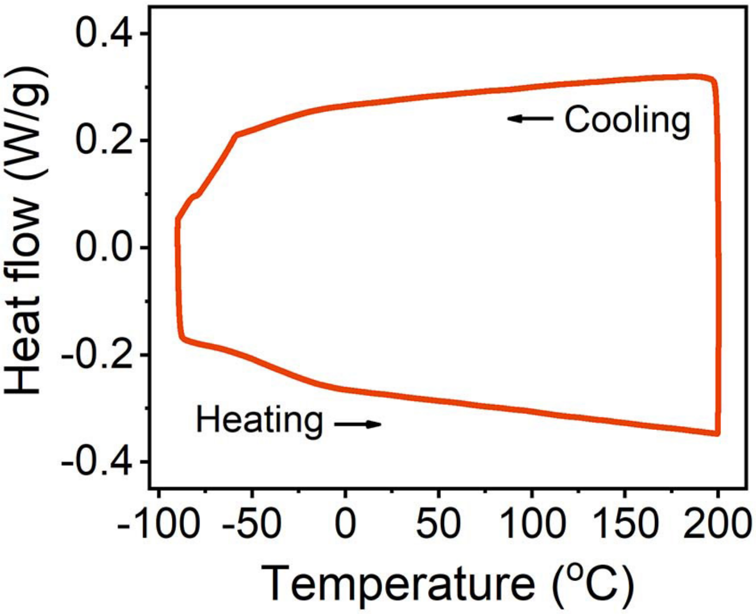

Figure S12. DSC curve of the MXene-free ionogel in a temperature range of -90 to 200 ºC.

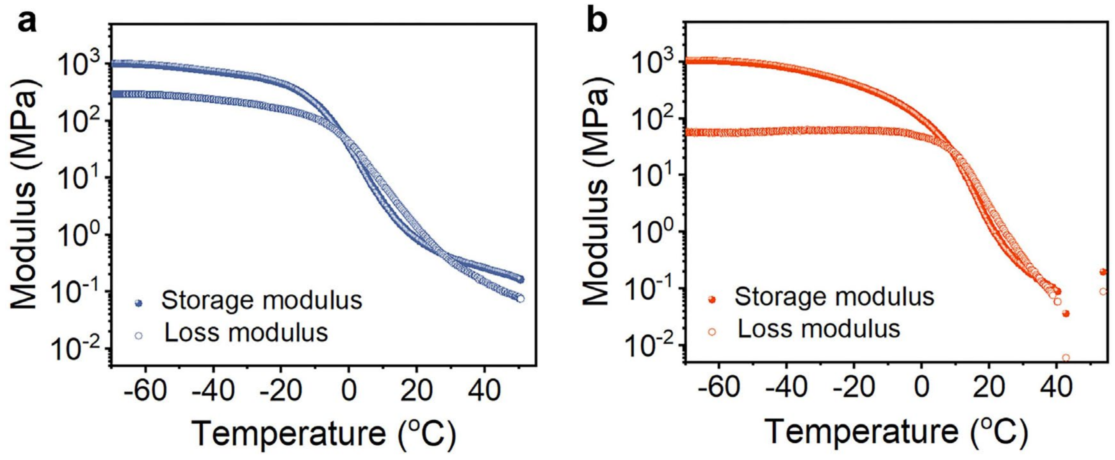

Figure S13. DMA curves of the ionogels (a) with and (b) without MXene in a temperature range of -70 to 55 ºC.

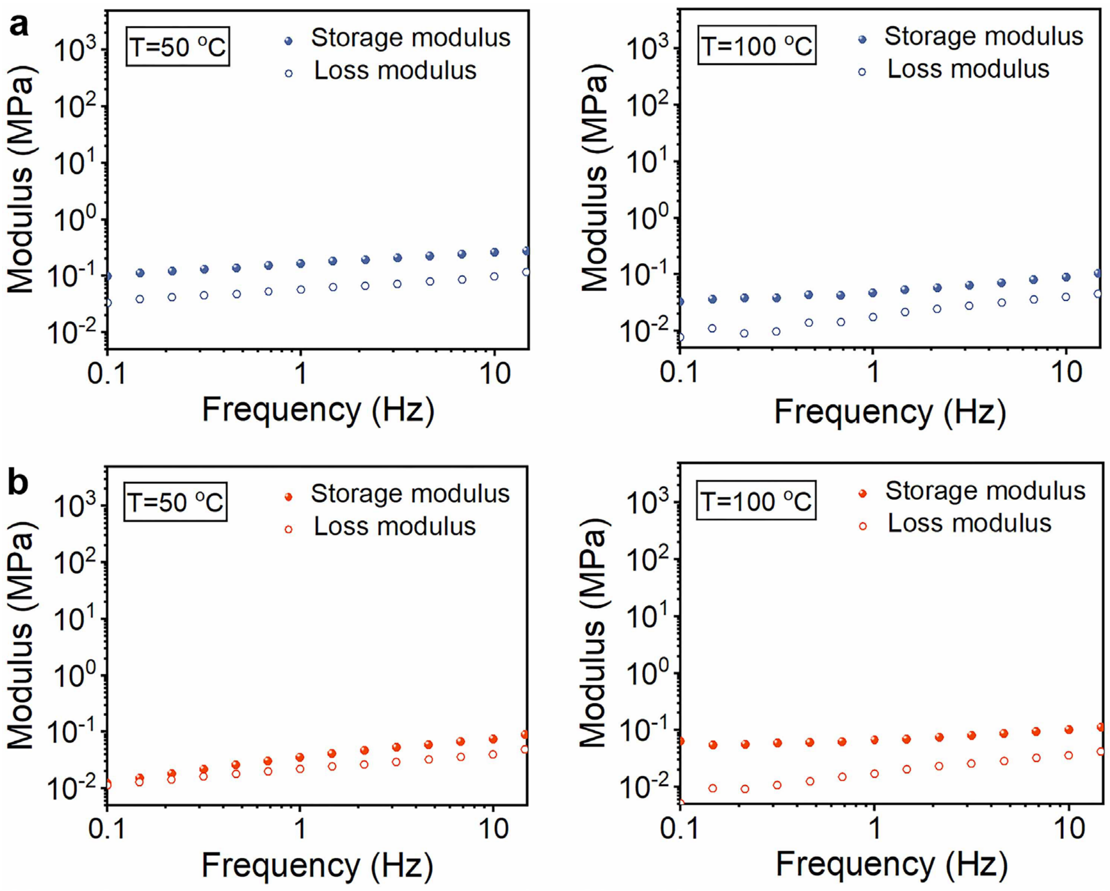

Figure S14. Rheological behaviors of the ionogels (a) with and (b) without MXene at the high temperature of 50 and 100 °C.

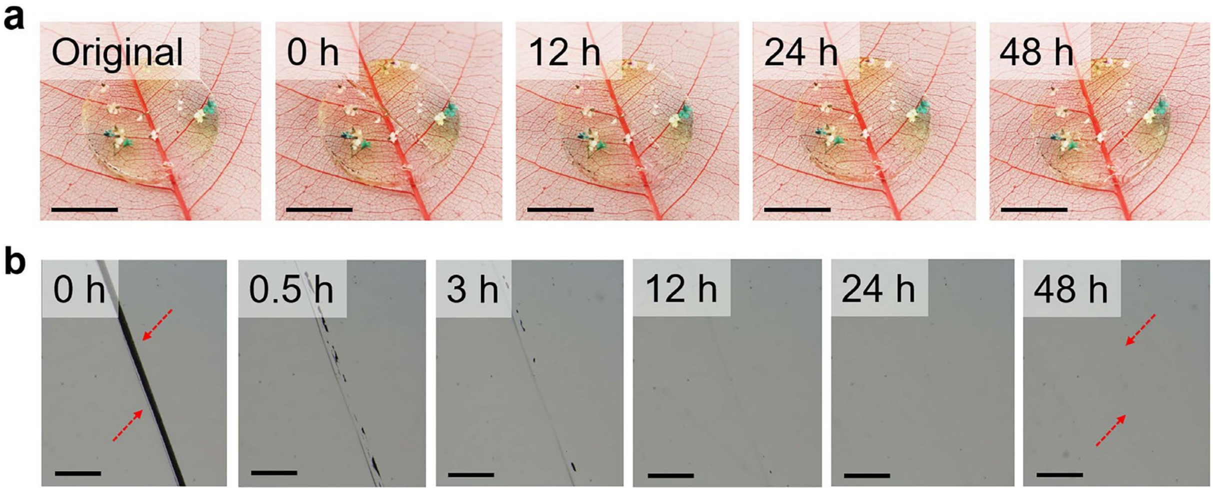

Figure S15. (a) Photographs and (b) optical micrographs of the autonomously self-healing process of the MXene-free ionogel. Scale bar: 1 cm and 500 μm, respectively.

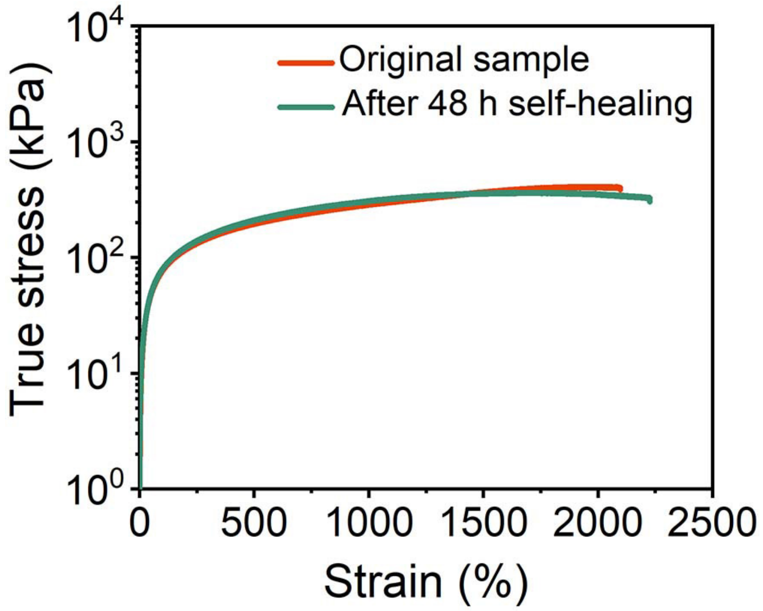

Figure S16. True tensile stress-strain curves of the original and healed ionogel without MXene.

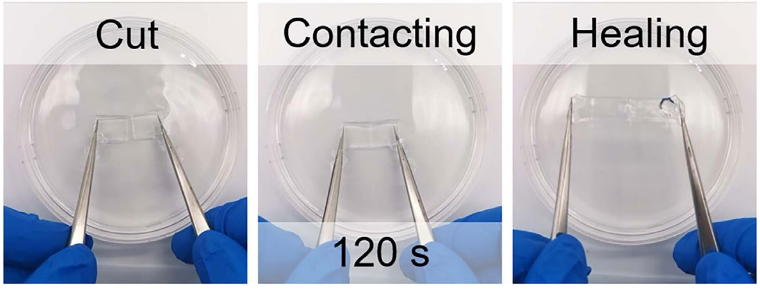

Figure S17. Photographs of the underwater self-healing process of the MXene-free ionogel.

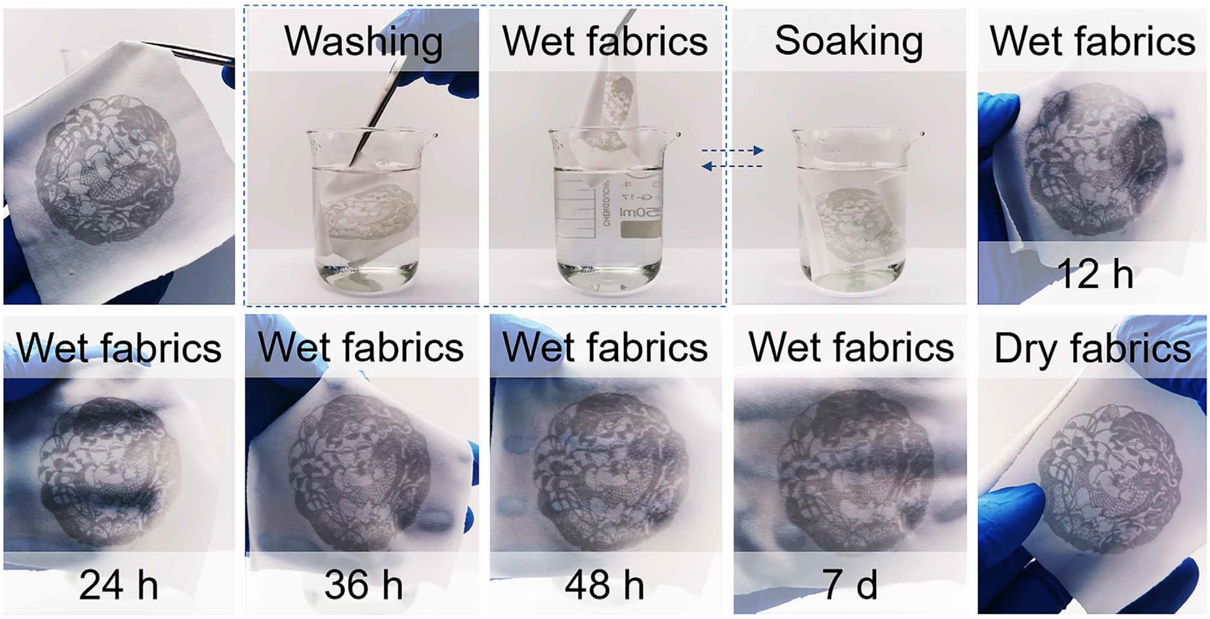

Figure S18. Underwater stability photographs of patterned fabrics for different periods following washing.

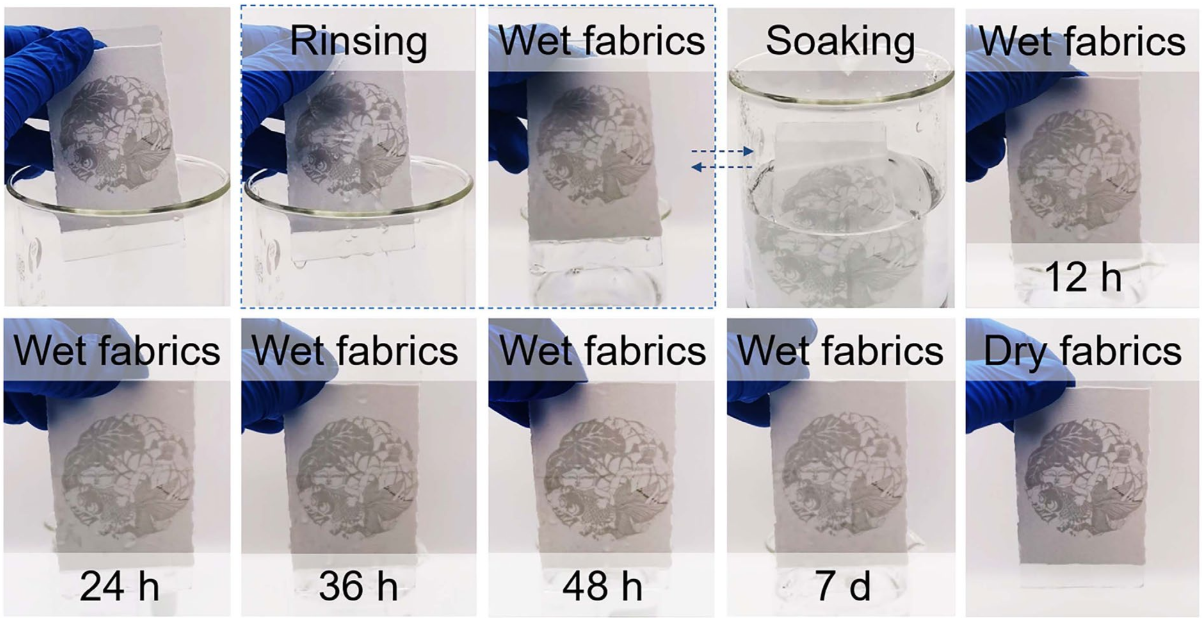

Figure S19. Underwater stability photographs of patterned fabrics for different periods following rinsing.

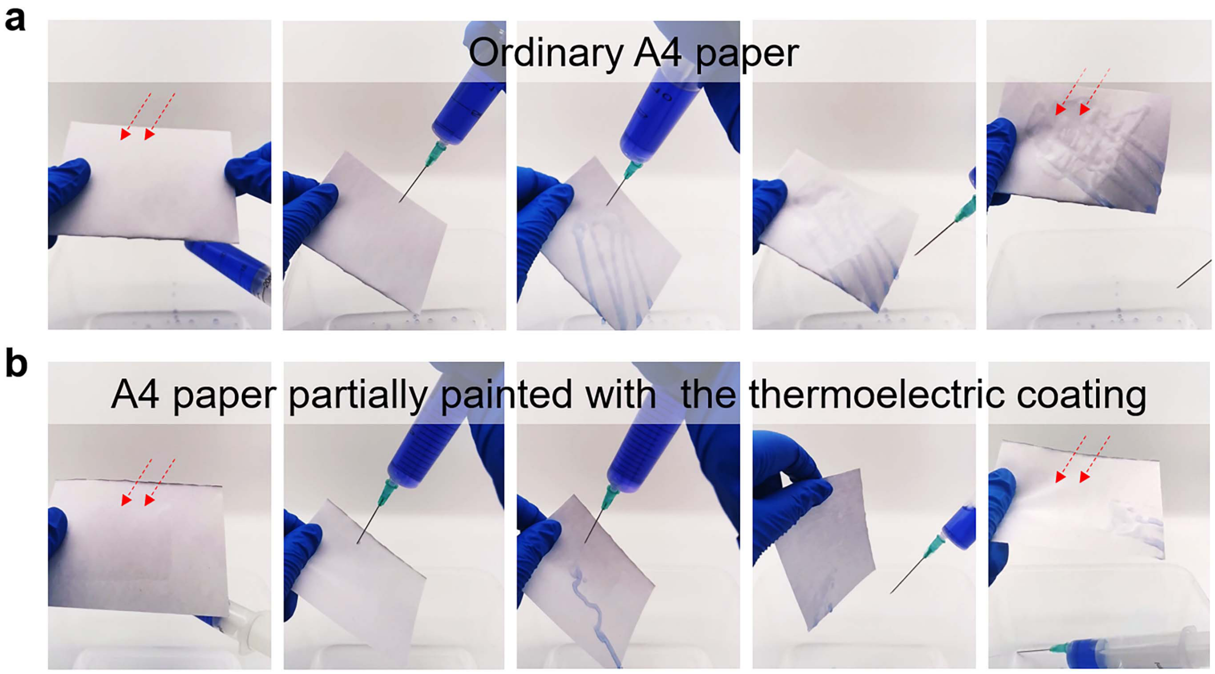

Figure S20. Comparison photographs of hydrophobicity for ordinary A4 paper (a) without and (b) with the MXene-free coating.

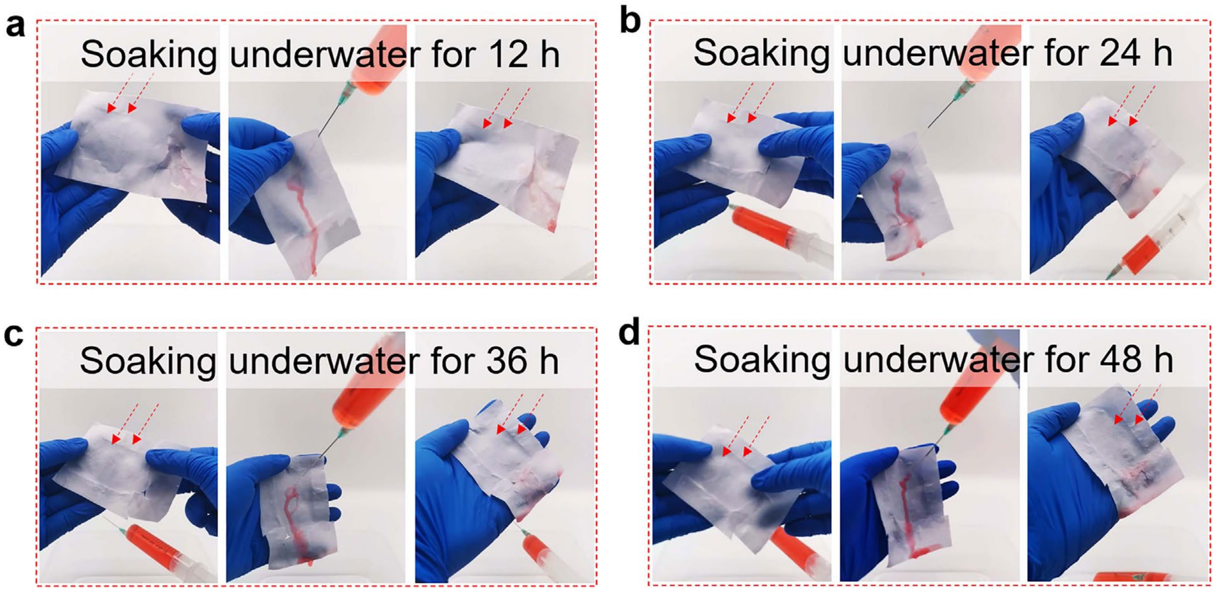

Figure S21. Comparison photographs of underwater stability for ordinary A4 paper with and without MXene-free coating.

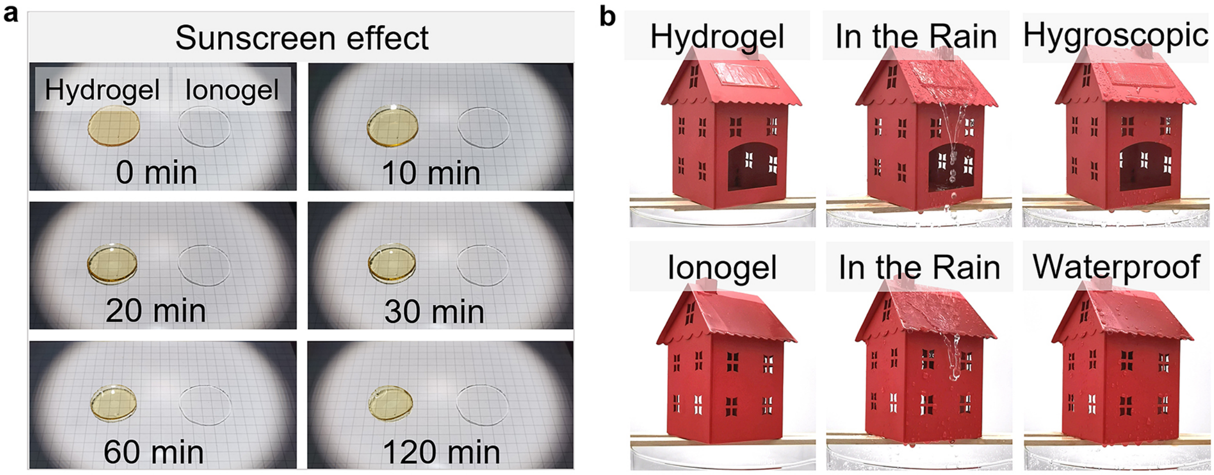

Figure S22. Comparison photographs of (a) sunscreen and (b) waterproof capability for PAM-PSSH hydrogel and the ionogels without/with MXene.

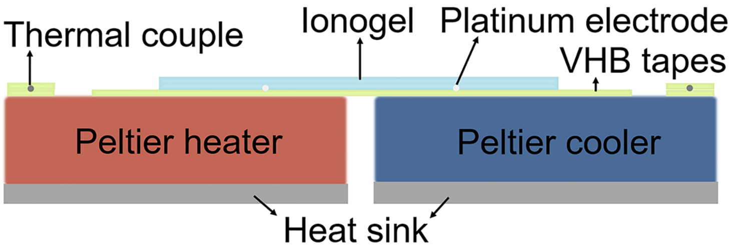

Figure S23. Schematic image of the self-made temperature gradient platform for the ionic Seebeck coefficient measurement.

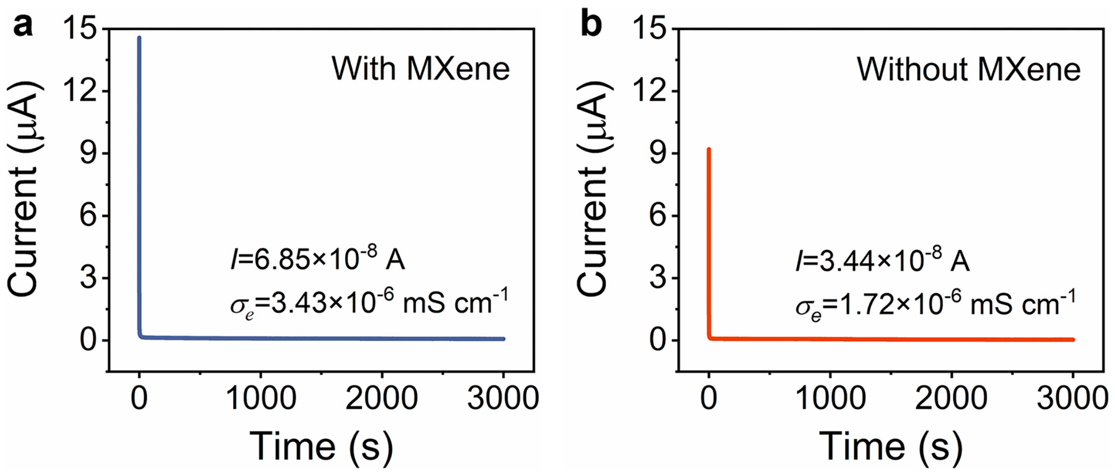

Figure S24. The polarization current-time curves of the ionogels with and without MXene at room temperature (293 K).

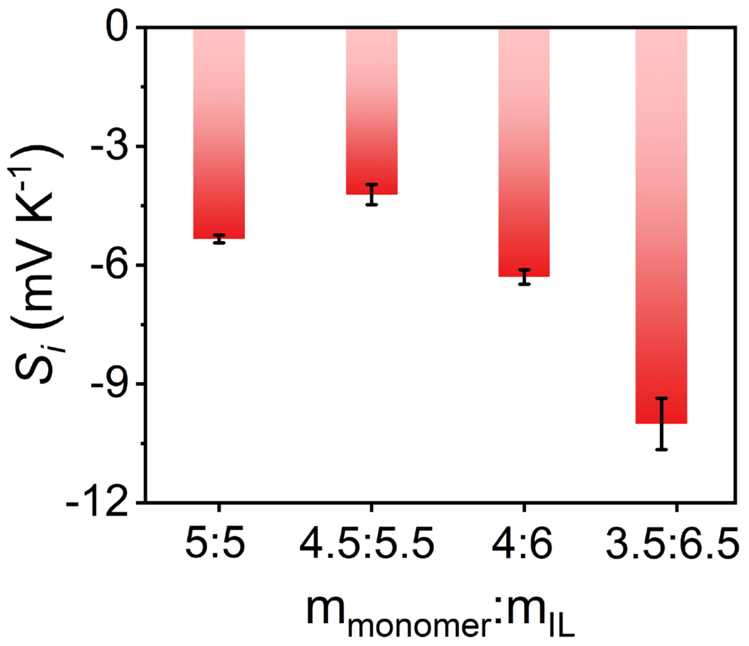

Figure S25. Ionic Seebeck coefficients (S_i) of the MXene-free ionogel with different weight ratios of monomer to IL. Data are presented as mean ± SD, n = 3.

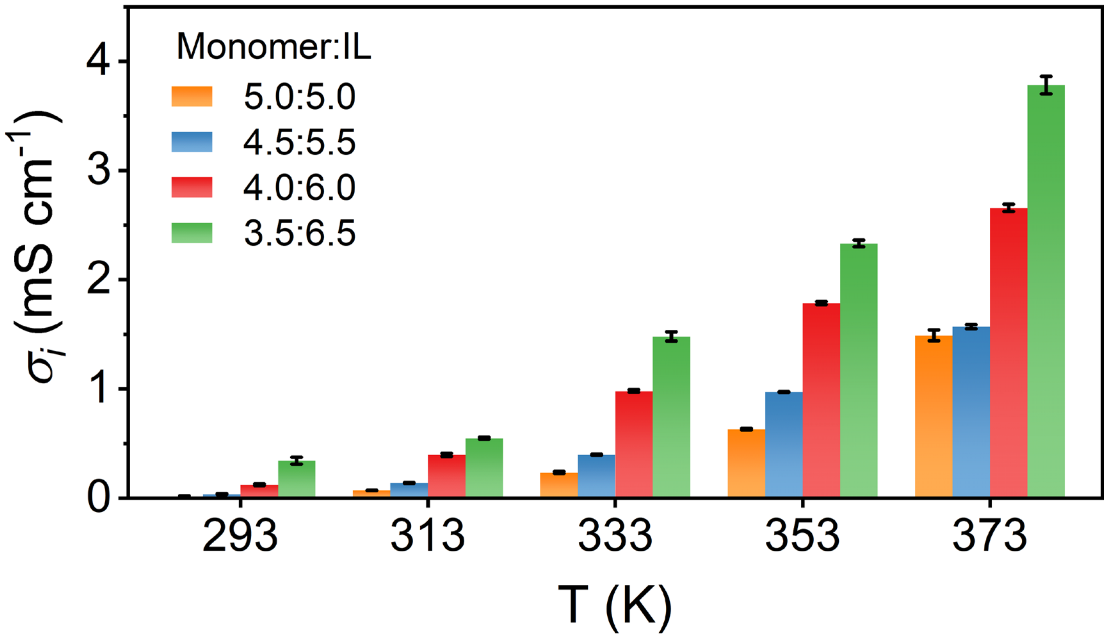

Figure S26. Conductivities (σ_i) of the MXene-free ionogel with various weight ratios of monomer to IL at different temperatures. Data are presented as mean ± SD, n = 3.

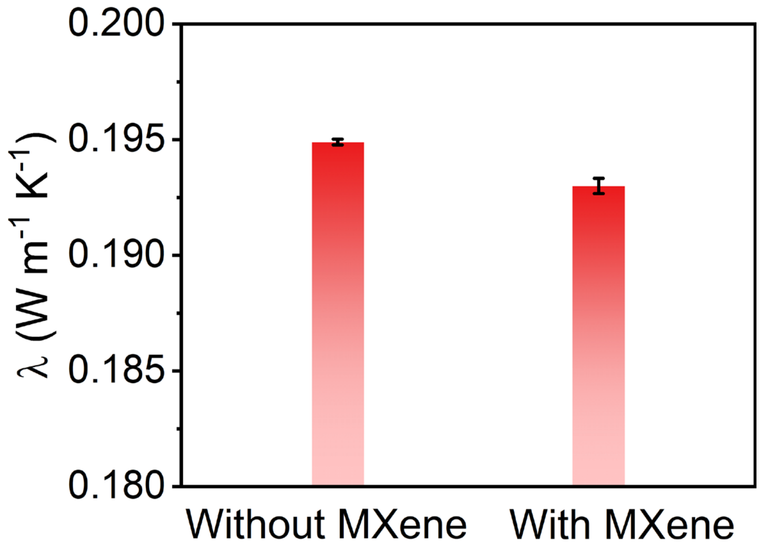

Figure S27. Thermal conductivity (λ) of the ionogels measured by a modified transient line source method. Data are presented as mean ± SD, n = 3.

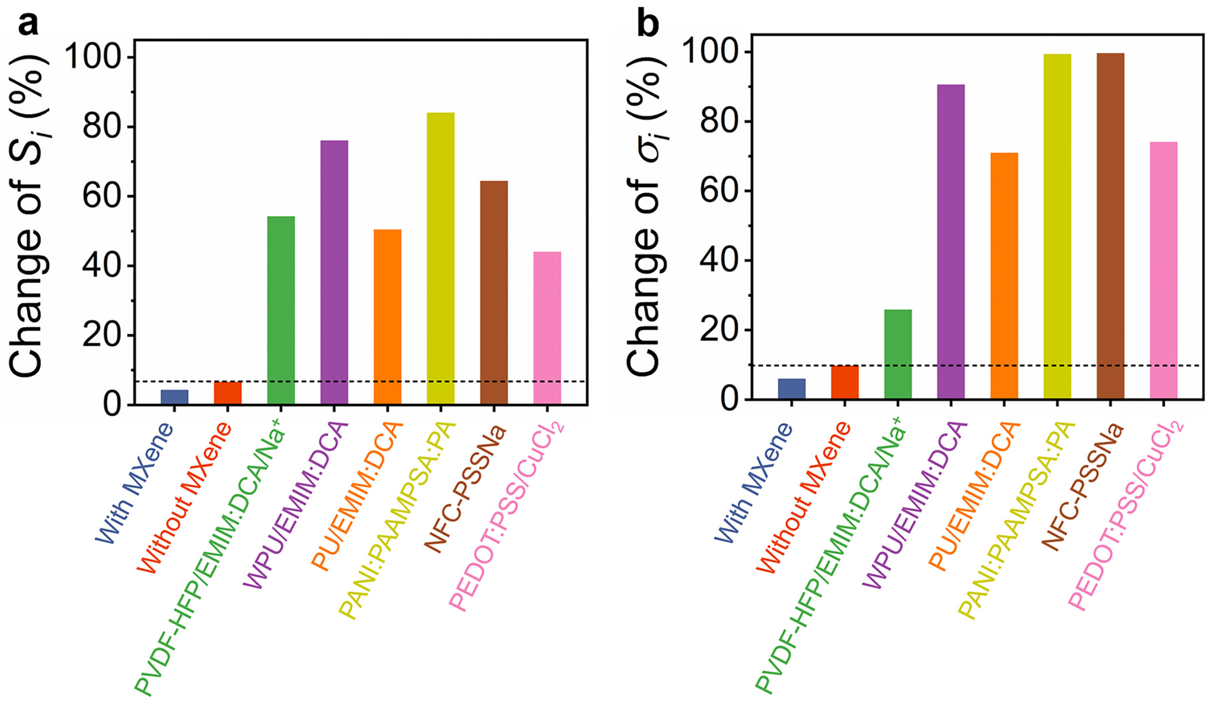

Figure S28. The stability comparison of thermoelectric properties (S_i, σ_i) between our work and previous thermoelectric ionogels/hydrogels when environmental humidity changes.

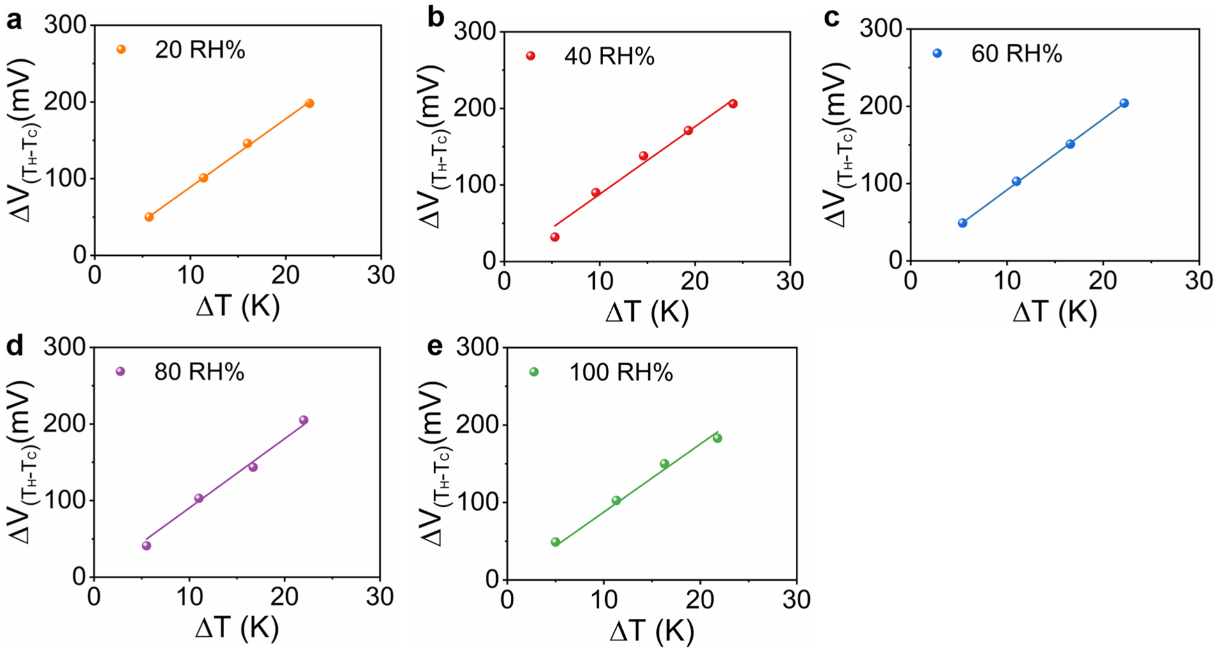

Figure S29. Potential difference at hot and cold terminals ($\Delta V_{(TH-TC)}$) of the ionogel with MXene under different temperature gradients (ΔT). The slope of the $\Delta V_{(TH-TC)}$-ΔT curve is the absolute value of the ionic Seebeck coefficient (S_i).

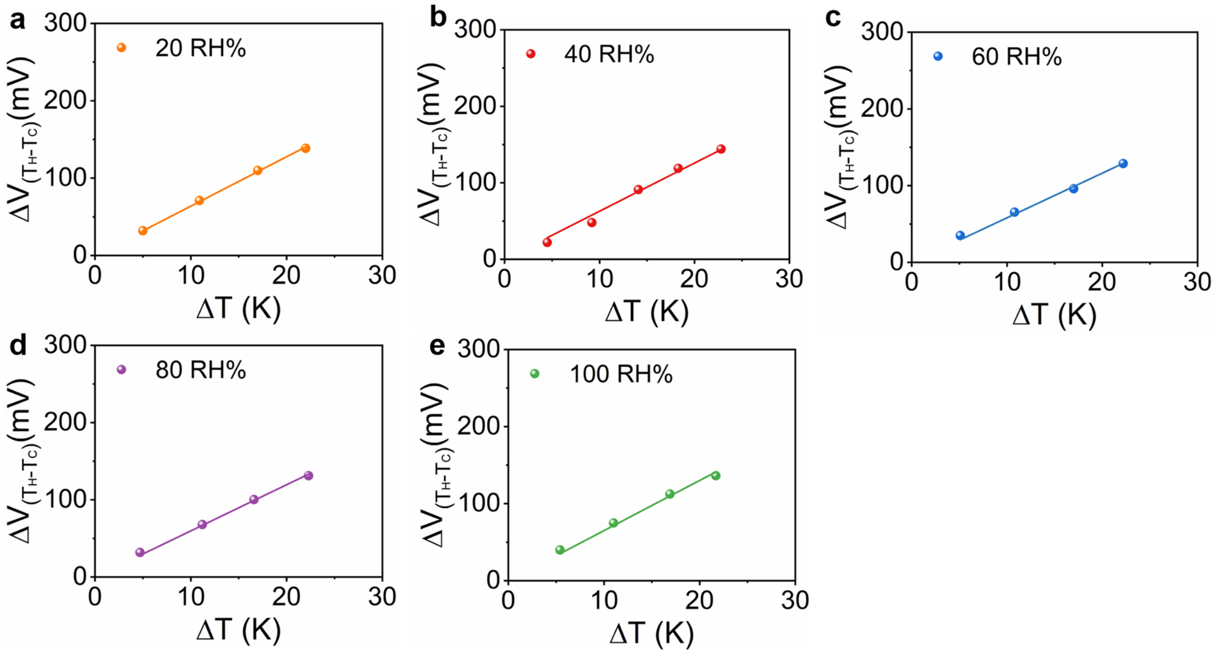

Figure S30. Potential difference at hot and cold terminals ($\Delta V_{(TH-TC)}$) of the ionogel without MXene under different temperature gradients (ΔT). The slope of the $\Delta V_{(TH-TC)}$-ΔT curve is the absolute value of the ionic Seebeck coefficient (S_i).

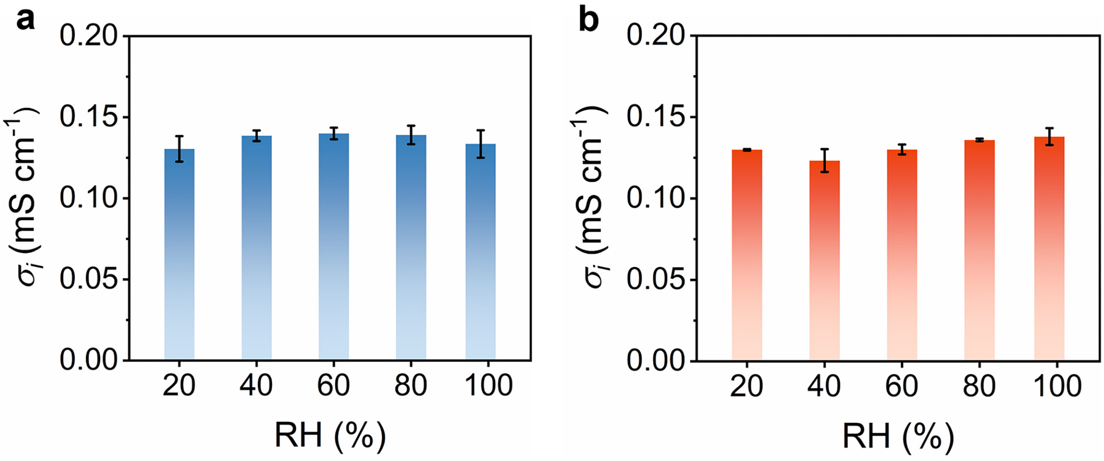

Figure S31. Conductivity (σ_i) changes of the ionogels (a) with and (b) without MXene under different relative humidities at 293 K. Data are presented as mean ± SD, n = 3.

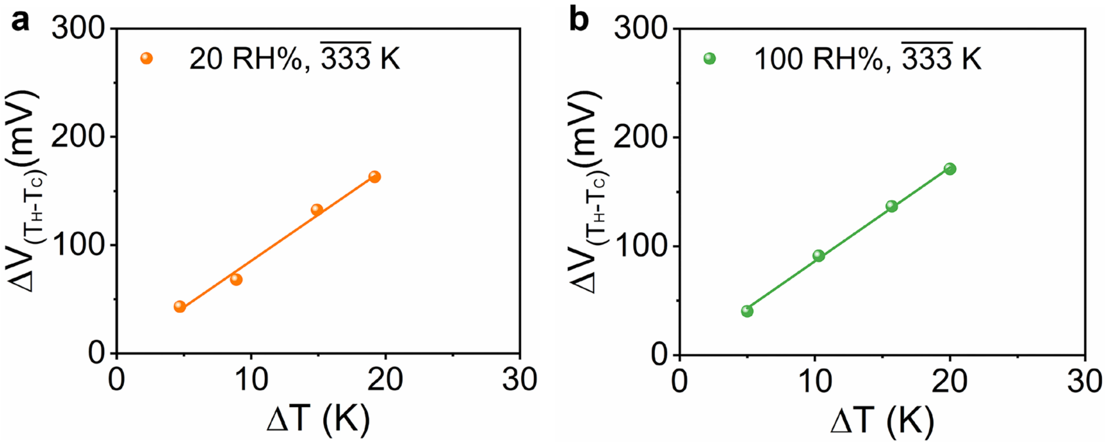

Figure S32. Potential difference at hot and cold terminals ($\Delta V_{(TH-TC)}$) of the ionogel with MXene under different temperature gradients (ΔT) at extreme average temperature (333 K) and relative humidities. The slope of the $\Delta V_{(TH-TC)}$-ΔT curve is the absolute value of the ionic Seebeck coefficient (S_i).

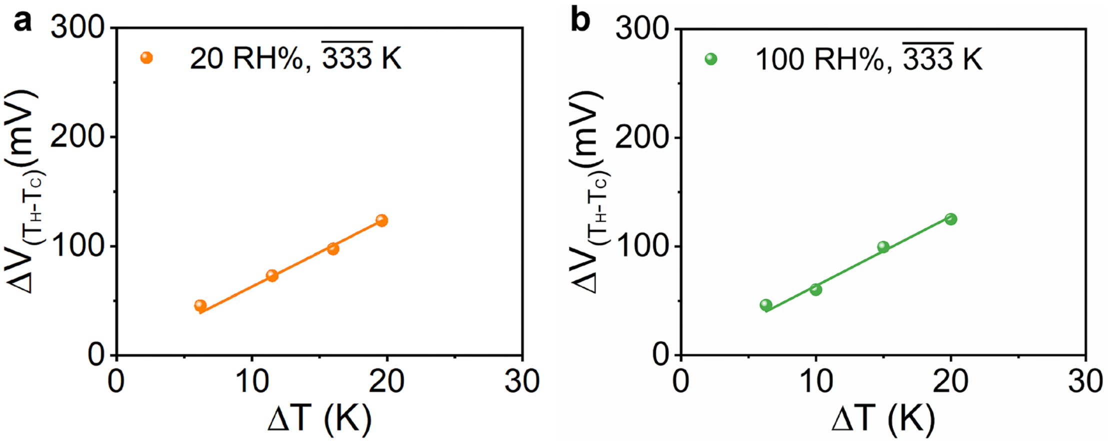

Figure S33. Potential difference at hot and cold terminals ($\Delta V_{(TH-TC)}$) of the MXene-free ionogel under different temperature gradients (ΔT) at extreme average temperature (333 K) and relative humidities. The slope of the $\Delta V_{(TH-TC)}$-ΔT curve is the absolute value of the ionic Seebeck coefficient (S_i).

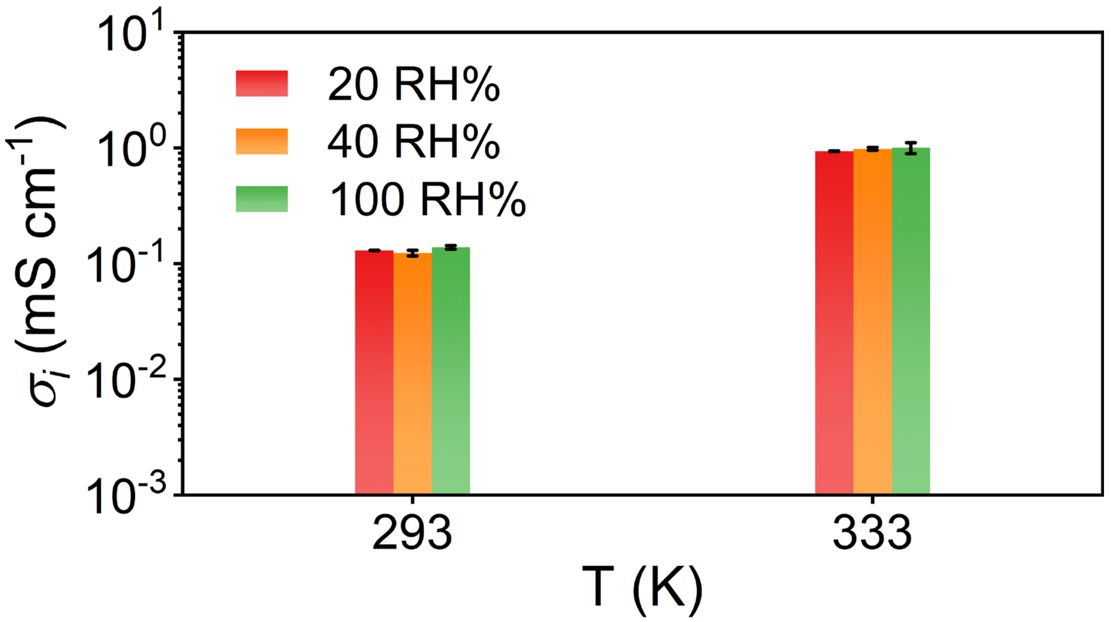

Figure S34. Conductivity (σ_i) changes of the MXene-free ionogels in wide temperature and humidity ranges (293-333 K, 20-100 RH%). Data are presented as mean ± SD, n = 3.

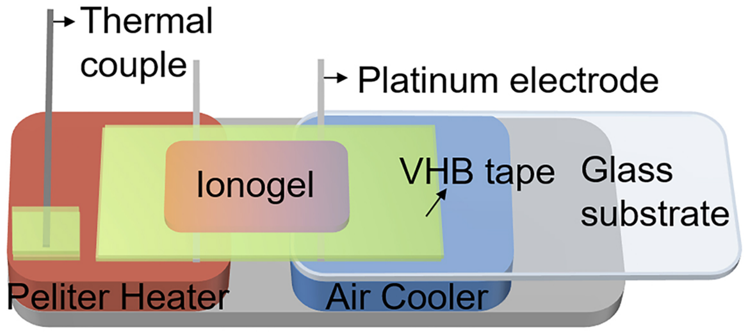

Figure S35. Schematic image of the self-made temperature gradient platform for the stability measurement of thermoelectric performance during repeated multiple deform (including bend, stretch and twist) -release processes.

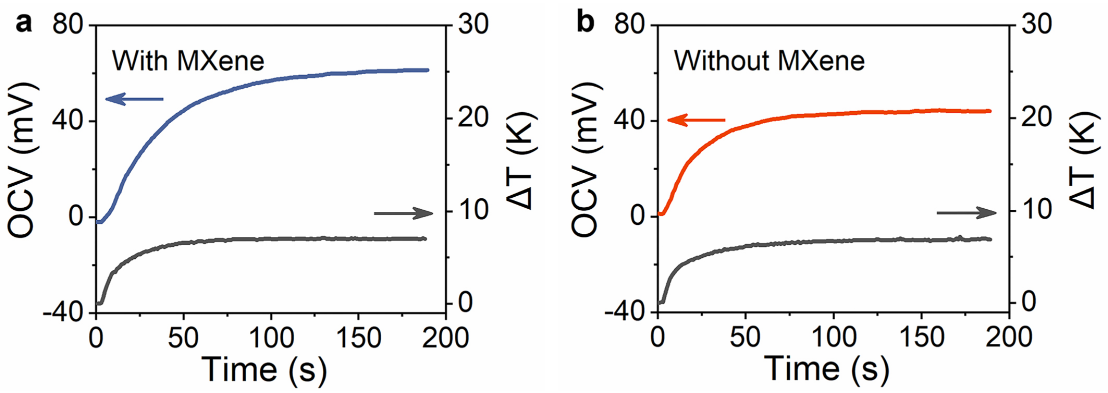

Figure S36. Open circuit voltage and temperature curves of the ionogels with and without MXene under a temperature gradient of 7 K.

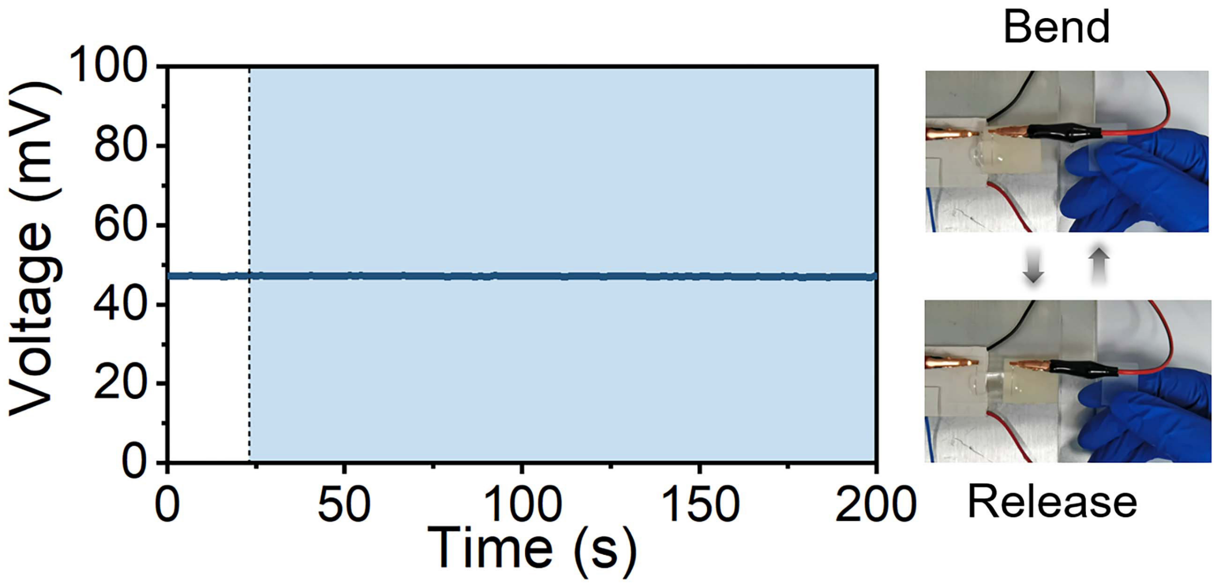

Figure S37. Voltage-time curve of the MXene-free ionogel being repeatedly step-bent and a manual bend-release photograph.

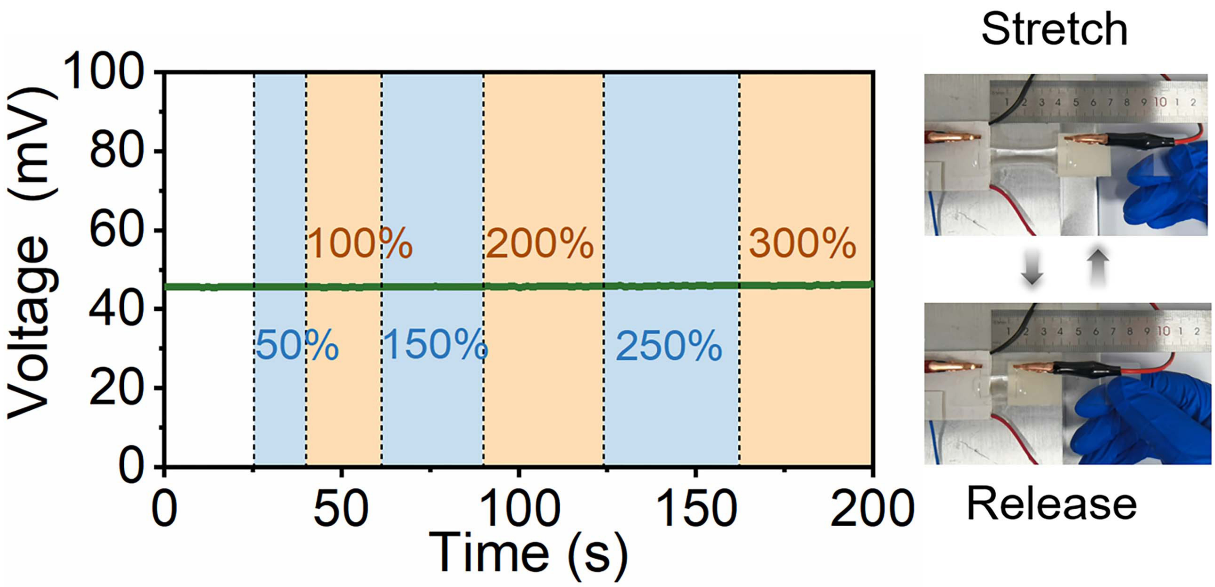

Figure S38. Voltage-time curve of the MXene-free ionogel being manually stretched from 50 to 300 % strain and a manual stretch-release photograph.

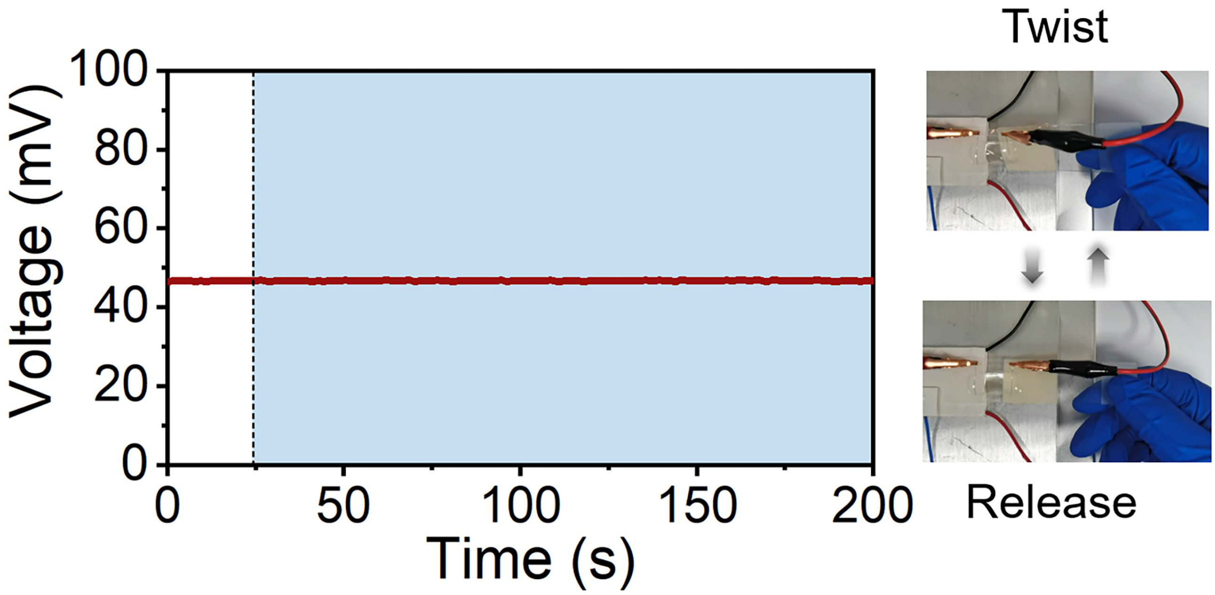

Figure S39. Voltage-time curve of the MXene-free ionogel being repeatedly twisted and a manual twist-release photograph.

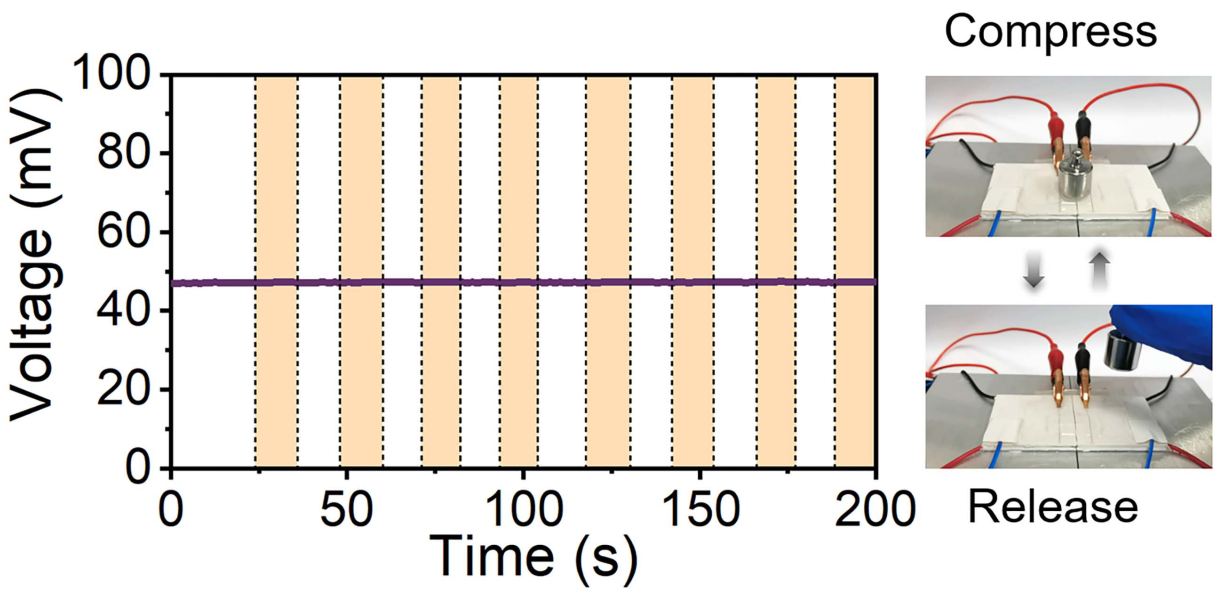

Figure S40. Voltage-time curve of the MXene-free ionogel being repeatedly compressed-released by a 20 g load and a corresponding photograph.

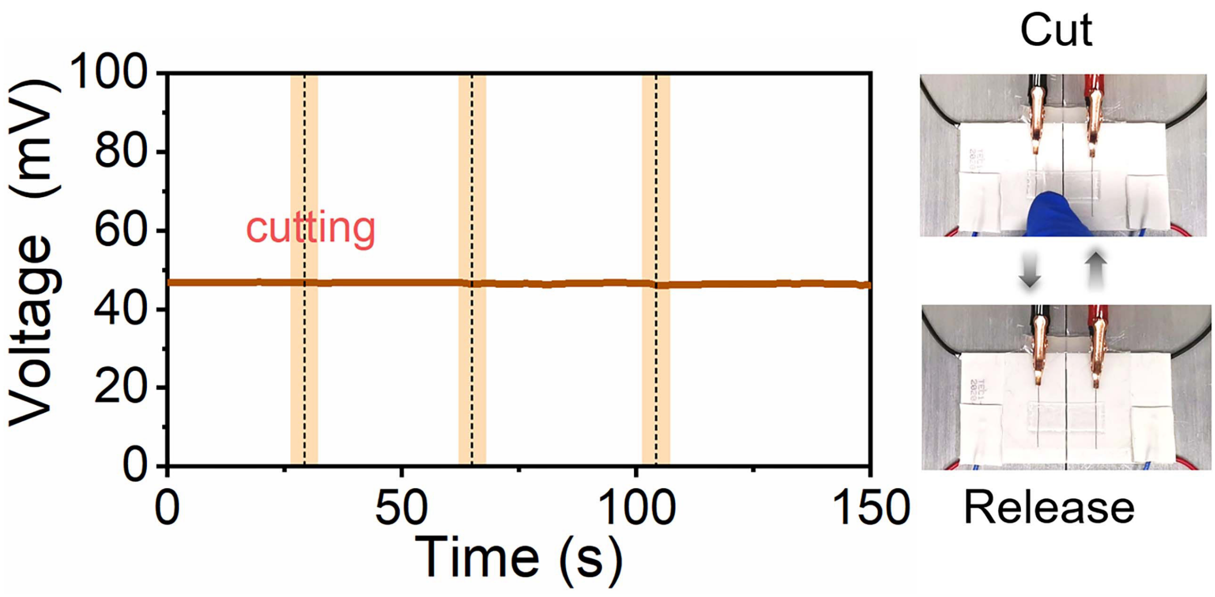

Figure S41. Voltage-time curve of the MXene-free ionogel being repeatedly cut by a sharp knife and a manual cut-release photograph.

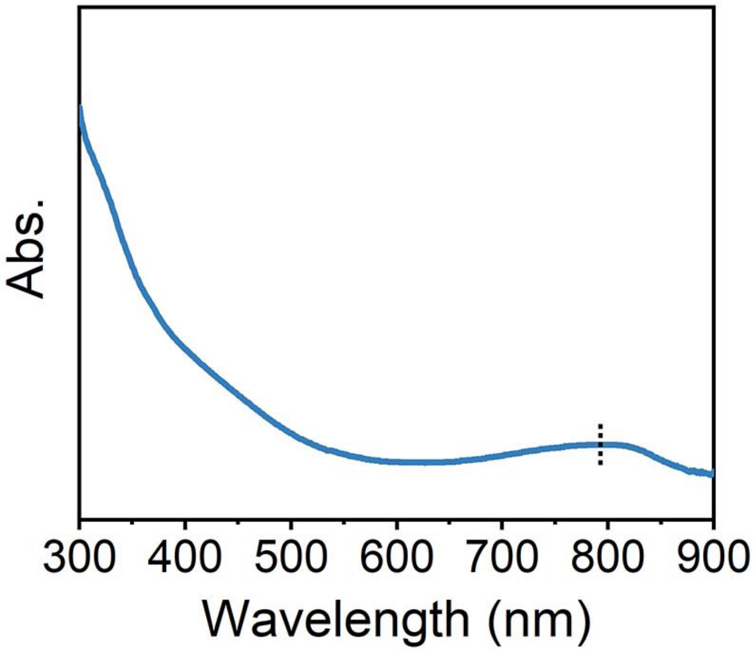

Figure S42. UV-vis absorption spectra of the ionogel with MXene.

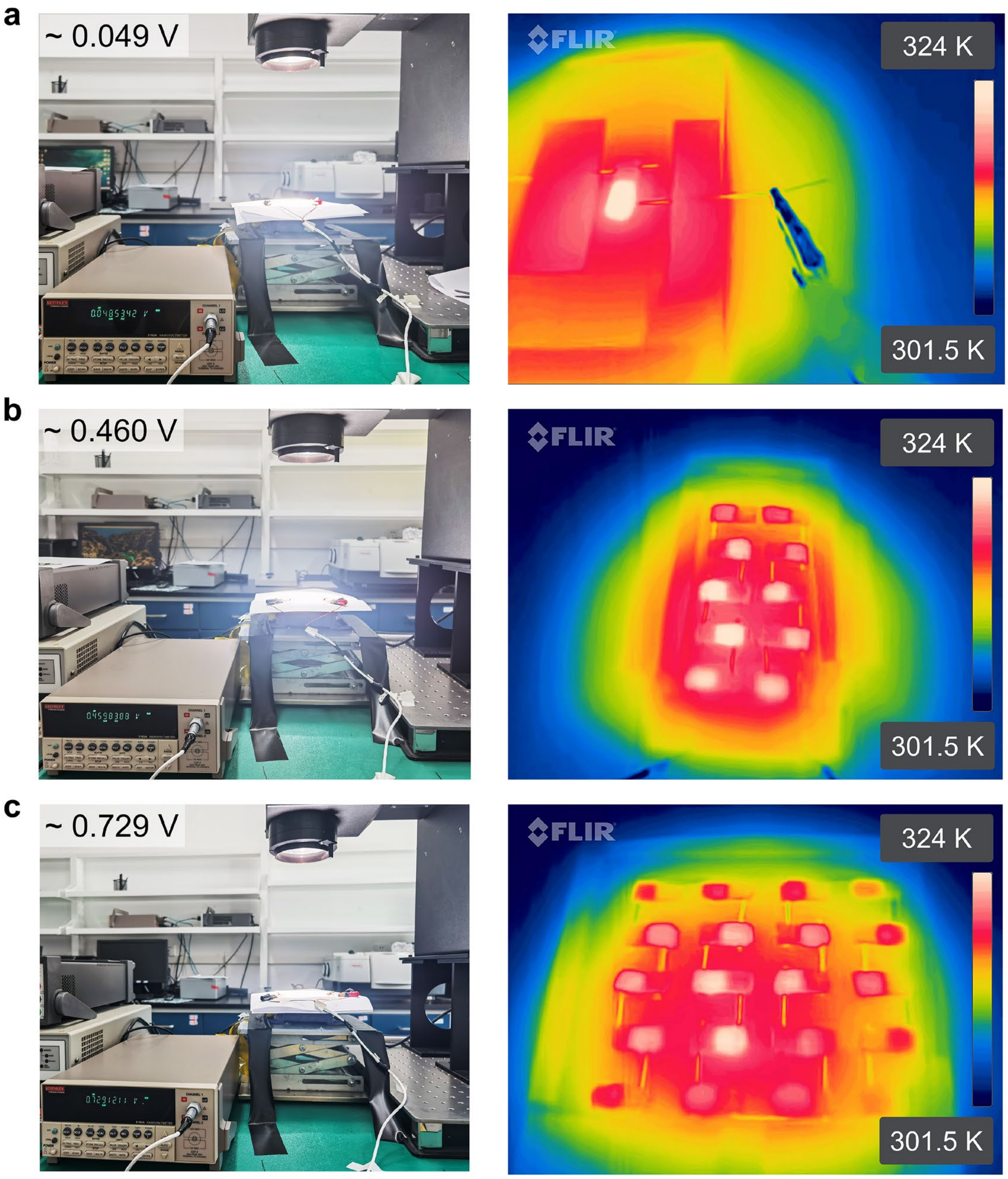

Figure S43. Open circuit thermovoltages and infrared images of different integrated arrays containing (a) 1, (b) 10, and (c) 20 units under one sun illumination with moderate intensity about 0.3 kW m^{-2}, respectively.

Table S1. The binding energies between molecules from DFT calculations.

Molecules	Binding energies (kcal mol^{-1})
MMA-MMA	691.27
MMA-MA	71.24
[EMIM][TFSI]	19.36
MMA-$[EMIM]^{+}$	475.56
MMA-$[TFSI]^{-}$	112.07
MA-$[EMIM]^{+}$	54.76
MA-$[TFSI]^{-}$	300.45
MA-MA	4.91

Table S2. The IR band migrations of P(MMA-co-MA), $[TFSI]^-$ anions, and $[EMIM]^+$cations from the ionogels.

<table>
<tr><th rowspan="2">Bands</th><th colspan="3">Wavenumber (cm^{-1})</th></tr>
<tr><th>Original positions</th><th colspan="2">Final positions</th></tr>
<tr><td rowspan="2">-C-H</td><td>2993</td><td colspan="2">2995</td></tr>
<tr><td>2952</td><td colspan="2">2955</td></tr>
<tr><td>-C=O</td><td>1722</td><td colspan="2">1727</td></tr>
<tr><td rowspan="2">-O=S=O-</td><td>1349</td><td colspan="2">1352</td></tr>
<tr><td>1325</td><td colspan="2">1327</td></tr>
<tr><td rowspan="2">$-CF_3$</td><td>1184</td><td colspan="2">1186</td></tr>
<tr><td>1167</td><td colspan="2">1172</td></tr>
<tr><td>-SNS-</td><td>1052</td><td colspan="2">1055</td></tr>
<tr><td rowspan="2">-C-H</td><td>3126</td><td>3123</td><td>3125</td></tr>
<tr><td>3101</td><td>3098</td><td>3100</td></tr>
</table>

Table S3. Comparation of water-proof property, self-healing property, fracture strain and thermoelectric parameters between our ionogels and other i-TE materials including ionogels, ionic conducting polymers, ionic quasi-solid and liquid (thermoelectrochemical cells) etc.

Category	Sample	Water-Proof	Self-healing	Fracture strain (%)	Type	S_i, mV K^{-1}	PF, mW m^{-1} K^{-2}
Ionogels	Our work	√	√	~ 1300	n	-8.8	2.5×10^{-2}
	PVDF-HFP /EMIM:TFSI ionogels[4]	√	—	—	n	-4.0	3.2×10^{-3}
	WPU/EMIM:DCA ionogels[5]	—	—	~ 156	p	15.0	3.6×10^{-2}
	PU/EMIM:DCA ionogels[6]	—	√	~ 300	p	15.6	5.1×10^{-2}
	PANI/PAAMPSA/PA films[7]	—	√	~ 750	p	1.3	2.8×10^{-4}
Ionic conducting polymers	NFC-PSSNa composite[8]	—	—	~ 10.2	p	3.0	3.6×10^{-5}
	AgOH-Nafion[9]	—	—	—	n	-2.0	—
	PEDOT:PSS/$InCl_3$ ionic conductors[10]	—	—	—	n	-9.63	—
	PEDOT:PSS/$CuCl_2$ composite[11]	—	—	—	n	-10.2	0.14
Ionic quasi-solid thermoelectro-chemical cells	P(AM-co-AMPS)-$Fe(CN)_6^{4-}$/$Fe(CN)_6^{3-}$ Thermocell[12]	—	—	~ 217	p	1.6	—
	PVA-$FeCl_2$/$FeCl_3$ thermocell[13]	—	—	~ 110	n	-0.85	—
	Ionic gelatin composite[14]	—	—	~ 200	p	17.0	—
Ionic liquid thermoelectro-chemical cells	Gdm^+-$Fe(CN)_6^{4-}$ /$Fe(CN)_6^{3-}$ liquid thermocell[15]	—	—	—	p	3.73	—
	α -CD-I^{3-}/I^--KCl liquid thermocell[16]	—	—	—	p	2.0	—

Table S4. Stability comparison of thermoelectric properties (S_i, σ_i) between our work and previous thermoelectric ionogels/hydrogels when environmental humidity changes.

Sample	Range of humidity (%)	Change of S_i (%)	Change of σ_i (%)
This work with MXene	20 ~ 100	4.2	5.8
This work without MXene	20 ~ 100	6.5	9.7
PVDF-HFP/EMIM:DCA/ Na^+[17]	55 ~ 85	54.2	25.8
WPU/EMIM:DCA[5]	30 ~ 90	75.9	90.5
PU/EMIM:DCA[6]	50 ~ 90	50.3	70.8
PANI:PAAMPSA:PA[7]	50 ~ 90	84.0	99.3
NFC-PSSNa[8]	50 ~ 100	64.3	99.6
PEDOT:PSS/$CuCl_2$[11]	60 ~ 80	44.0	74.0

References

[1] H. Cheng, X. He, Z. Fan, and J. Ouyang, *Adv. Energy Mater.* **2019**, *9*, 1901085.
[2] F. Wu, N. Chen, R. Chen, Q. Zhu, J. Qian, and L. Li, *Chem. Mater.*, **2016**, *28*, 848.
[3] Y. Hao, D. Feng, L. Hou, T. Li, Y. Jiao, P. Wu, *Adv. Sci.*, **2022**, *9*, 2104832.
[4] D. Zhao, A. Martinelli, A. Willfahrt, T. Fischer, D. Bernin, Z. U. Khan, M. Shahi, J. Brill, M. P. Jonsson, S. Fabiano, *Nat. Commun.*, **2019**, *10*, 1093.
[5] Y. Fang, H. Cheng, H. He, S. Wang, J. Li, S. Yue, L. Zhang, Z. Du, J. Ouyang, *Adv. Funct. Mater.*, **2020**, *30*, 2004699.
[6] J. Xu, H. Wang, X. Du, X. Cheng, Z. Du, H. Wang, *ACS Appl. Mater. Interfaces* **2021**, *13*, 20427.
[7] Z. A. Akbar, J. W. Jeon, S. Y. Jang, *Energy Environ. Sci.*, **2020**, *13*, 2915.
[8] F. Jiao, A. Naderi, D. Zhao, J. Schlueter, M. Shahi, J. Sundstrom, H. Granberg, J. Edberg, U. Ail, J. Brill, T. Lindstrom, M. Berggrena and X. Crispin, *J. Mater. Chem. A*, **2017**, *5*, 16883.
[9] W.B. Chang, C.M. Evans, B.C. Popere, B.M. Russ, J. Liu, J. Newman, R.A. Segalman, *ACS Macro Lett.* **2016**, *5*, 94.
[10] Y. Shu, G. O. Odunmbaku, Y. He, Y. Zhou, H. Cheng, J. Ouyang, K. Sun, *Appl. Phys. Lett.*, **2021**, *118*, 103902.
[11] B. Kim, J. U. Hwang, E. Kim, *Energy Environ. Sci.*, **2020**, *13*, 859.
[12] Z. Lei, W. Gao, P. Wu, *Joule* **2021**, *5*, 2211.
[13] Y. Liu, S. Zhang, Y. Zhou, M. A. Buckingham, L. Aldous, P. C. Sherrell, G. G. Wallace, G. Ryder, S. Faisal, D. L. Officer, S. Beirne, J. Chen, *Adv. Energy Mater.*, **2020**, *10*, 2002539.
[14] C.-G. Han, X. Qian, Q. Li, B. Deng, Y. Zhu, Z. Han, W. Zhang, W. Wang, S.-P. Feng, G. Chen, *Science* **2020**, *368*, 1091.
[15] B. Yu, J. Duan, H. Cong, W. Xie, R. Liu, X. Zhuang, H. Wang, B. Qi, M. Xu, Z. L. Wang, J. Zhou, *Science*, **2020**, *370*, 342.
[16] H. Zhou, T Yamada, N. Kimizuka, *J. Am. Chem. Soc.*, **2016**, 138, 10502.
[17] Z. Liu, H. Cheng, Q. Le, R. Chen, J. Li, J. Ouyang, *Adv. Energy Mater.*, **2022**, *12*, 2200858.
